# Pan-cancer analysis of the prognostic and immunological role of PSMB8

**DOI:** 10.1038/s41598-021-99724-9

**Published:** 2021-10-14

**Authors:** Danxiang Chen, Cong Jin, Xubin Dong, Jialiang Wen, Erjie Xia, Qingxuan Wang, Ouchen Wang

**Affiliations:** Department of Breast Surgery, The First Affiliated Hospital of Wenzhou Medical University, Nan Bai Xiang Street, Wenzhou, 325006 Zhejiang China

**Keywords:** Cancer, Immunology, Molecular biology, Biomarkers, Oncology

## Abstract

Recently some evidence has demonstrated the significance of PSMB8 in various malignancies. Nevertheless, PSMB8 (proteasome subunit beta 8), more familiar in the field of immunology contributing to the process of antigen presentation, is indeterminate in the role as a survival predictor of human pan-cancer. Besides, how PSMB8 interacts with immune cell infiltration in the tumor microenvironment requires further research. We then penetrated into the analysis of the PSMB8 expression profile among 33 types of cancer in the TCGA database. The results show that overexpression of PSMB8 was associated with poor clinical outcomes in overall survival (Sartorius et al. in Oncogene 35(22):2881–2892, 2016), disease-specific survival (DSS), disease-free interval (DFI), and progression-free interval (PFI) in most cancer varieties. In addition, there existed distinctly positive correlations between PSMB8 and immunity, reflected straightforwardly in the form of immune scores, tumor-infiltrating immune cells (TIICs) abundance, microsatellite instability, tumor mutation burden, and neoantigen level. Notably, specific markers of dendrite cells exhibited the tightest association with PSMB8 expression in terms of tumor-related immune infiltration patterns. Moreover, gene enrichment analysis showed that elevated PSMB8 expression was related to multiple immune-related pathways. We finally validated the PSMB8 expression in our local breast samples via quantitative PCR assays and concluded that PSMB8 appeared to perform well in predicting the survival outcome of BRCA patients. These findings elucidate the pivotal role of the antigen presentation-related gene PSMB8, which could potentially serve as a robust biomarker for prognosis determination in multiple cancers.

## Introduction

Currently, several checkpoint-blocking drugs such as anti-CTLA4 and anti-PD-L1 have provided superior performance compared to conventional cytotoxic drugs, which has fueled the field of immune-related therapeutic targets in oncology. Considering the recent advances in immunotherapy, it is impossible to ignore the substantial influence of the tumor microenvironment (TME), which is comprised of resident stromal cells and recruited immune cells. The antigen-presenting system, in concert with immune effector cells, orchestrate sustained input to the TME-tumor interaction via the enhancement of tumor immunogenicity and the modulation of an anti-tumor microenvironment.

The PSMB8 gene encodes an essential subunit of a specialized immunoproteasome complex^1^. And the generated peptides has higher affinity with major histocompatibility complex (MHC) I molecules and in turn enhanced antigenicity to CD8+ T cells^2–4^. Meanwhile, PSMB8 mutations have been observed to contribute to auto-inflammation and lipodystrophy in humans^1^, which show the pleiotropic functions involved in the maintenance of dynamic equilibrium. Previous studies have revealed the context-specific role of PSMB8, which varies in diverse cancers. Recent studies have recognized a neovascularization-suppressive role exerted by PSMB8 in glioma, via modulation of the ERK1/2 and PI3K/AKT signaling pathways^5,6^. In the orthotopic mouse model, inducible knock-down of PSMB8 dampened the expression of vascular endothelial growth factor (VEGF) and CD31; and thus, favored invasive capacity in glioblastoma. In another functional analysis, the role of PSMB8 was recapitulated in mucinous ovarian carcinoma pathogenesis, which identified PSMB8 as a mediator between antigen presentation of exogenous antigen via MHC class I molecules and the noncanonical nuclear factor kappa-light-chain-enhancer of activated B cells (NIK/NF-kB) pathway^7^. Furthermore, PSMB8 meditated PI3K/AKT pathway activation in acute myeloid leukemia (LAML) was established and was suggested as a promotor of tumorigenesis^8^. In addition to the previously mentioned tumors, a large-scale public database analysis and laboratory investigations have successively confirmed the role of PSMB8 in the evolution of cutaneous squamous cell carcinoma, papillary thyroid carcinoma, and prostate adenocarcinoma^9–11^. The association of aberrant expression of PSMB8 have been asynchronous with regard to tumor prognosis. In contrast, there is also a growing body of research lending support to a shielding role for PSMB8 via the promotion of immune cell infiltration. In T cell-mediated anti-tumor immunity^12^, the overexpression of PSMB8 was reported to reduce colony formation after radiation with a significant increase in expression of apoptosis-inducing molecules, such as cleaved PARP and cleaved caspase-3^13^. To date, the mechanisms that underline the tumorigenesis capacity of PSMB8 are not fully understood, and the immunological and prognostic roles of PSMB8 in the pan-cancer background remain to be elucidated.

Previously published studies conducting the cancer-associated analysis involving PSMB8 have been limited to specific cancer type. Herein, we present a comprehensive evaluation of the immune-related prognostic landscape of PSMB8 in the pan-cancer field pooling information from publicly available databases. Our study was exploratory and interpretative in nature, and required a longitudinal analysis. In detail, we first compared the expression of PSMB8 in normal tissues, tumor cell lines, and pan-cancer. Transcriptome-sequencing patterns were conventionally followed by survival analysis. Subsequently we evaluated the prognostic value of PSMB8 in pan-cancer using datasets from The Cancer Genome Atlas (TCGA) database. Next, we analyzed the association between PSMB8 expression and the degree of immune cell infiltration, immune checkpoint expression, and mutational burden. To this end, we have shifted the research focus of PSMB8 toward a multi-dimensional analysis of clinical relevance, with an unique immuno-correlation study based on pan-cancer analysis. This study sheds light on the potential role of PSMB8 as a prognostic-indicator in different cancers.

## Materials and methods

### Data acquisition and processing

TCGA, a landmark cancer genomic database containing vast information on cancer samples spanning 33 cancer types, was exploited to extract expression profile data from matched tumor and adjacent normal samples, as well as information detailing the corresponding clinicopathological traits. Another comprehensive public resource, GTEx, was applied to enrich tissue-specific normal samples alongside those obtained from TCGA. The broad institute CCLE was interrogated for PSMB8 mRNA expression in human cancer cell lines for a multi-dimensional inspection of PSMB8 expression.

Prior to commencing the study, the transcript data were checked for their robustness and normalization. Subsequently, the RNA sequencing data were adjusted to eliminate missing and duplicated results, and were transformed by a log2(TPM + 1) normalization using the R package of "rma" in an R environment (R version: 3.6.1). Cases in pan-cancer were acquired having a thorough follow-up for survival analysis and their corresponding outcomes in terms of overall survival^14^, disease-specific survival (DSS), and in the disease-free interval (DFI) and progression-free interval (PFI).

### PSMB8 gene expression analysis and correlation with malignancy prognosis

The recruited samples from TCGA in the section of the survival analysis were preliminarily screened for their data integrity in terms of both PSMB8 expression and follow-up information. Consequently, a prognosis-correlated analysis of the respective outcome events of OS, DSS, DFI, PFI was conducted. The optimal PSMB8 cut-off values for Kaplan–Meier curves were calculated using the function "survcutpoint" of "survminer" package in R (R version: 3.6.1). Kaplan–Meier curves and log-rank tests were used to estimate the prognosis-predictive value of PSMB8. Hazard ratios (HRs) with 95% confidence intervals and log-rank *P*s were calculated. HRs with a value less than 1 were detrimental to survival, while a value greater than 1 were beneficial to prognosis.

### TIMER analysis and immune microenvironment correlation analysis

To investigate the interplay between tumor and the TME, an immune correlation analysis was carried out. First, the TIMER (Tumor Immune Estimation Resource) web server (https://cistrome.shinyapps.io/timer/) was used to visualize the immune infiltration-associated expression pattern of PSMB8 in diverse cancer types. In the gene module of the TIMER database, we investigated the levels of PSMB8 expression and the abundance of tumor-infiltrating immune cells (TIICs), including CD4+ T cells, CD8+ T cells, B cells, neutrophils, dendritic cells (DCs), and macrophages. The scatter plots displayed purity-corrected partial Spearman’s rho values and statistical significances evaluated by the Wilcoxon test. In addition, the ESTIMATE algorithm was exploited to infer the ratio of immune and stromal components from PSMB8 expression data in pan-cancer, whose results were presented in the form of and immune score, stromal score, and ESTIMATE score with the relative correlation coefficients. Following the application of these immune algorithms, immune-stimulatory and immunoinhibitory gene markers were gathered for additional correlation analysis in pan-cancer, which comprised BLTA, CD200, TNFRSF14, NRP1, LAIR1, TNFSF4, CD244, LAG3, ICOS, CD40LG, CTLA4, CD48, CD28, CD200R1, HAVCR2, ADORA2A, CD276, KIR3DL1, CD80, PDCD1, LGALS9, CD160, TNFSF14, IDO2, ICOSLG, TMIGD2, VTCN1, IDO1, PDCD1LG2, HHLA2, TNFSF18, BTNL2, CD70, TNFSF9, TNFRSF8, CD27, TNFRSF25, VSIR, TNFRSF4, CD40, TNFRSF18, TNFSF15, TIGIT, CD274, CD86, CD44, and TNFRSF9. To further evaluate the immune-predictor value of PSMB8 in pan-cancer, we evaluated two emerging parameters—tumor mutation burden (TMB) and microsatellite instability (MSI)—in the context of immunotherapy. We proposed that TMB and MSI-associated PSMB8 expression would provide evidence that a higher degree of genomic instability, as defined by the TMB and MSI, could extrapolate to more immune-surveillance opportunities. Furthermore, the relationship between different immune checkpoint genes and PSMB8 expression were analyzed via correlation coefficients.

### Gene set enrichment analysis

To reflect the underlying biological function of PSMB8, we subsequently anatomized the biological traits by applying GSEA. Genes enriched in the predefined gene sets in GSEA (http://software.broadinstitute.org) served as the reference to assess the overall coupling of aberrantly-expressed PSMB8 enriched in the KEGG and HALLMARK collections, respectively. The significantly enriched pathways were finally identified based on the calculated net enrichment score (NES) with a false discovery rate (FDR) < 0.05 as the cut-off criterion.

### Association analysis of PSMB8 With DNA mismatch repair (MMR) genes and methyltransferases

DNA mismatch repair is an essential intracellular repair mechanism, which leads to the risk reduction of genomic instability. DNA methylation is a form of epigenetic modification that does not alter the DNA sequence. The correlation of five MMRs genes (MLH1, MSH2, MSH6, PMS2, EPCAM) as well as four methyltransferases with PSMB8 expression was assessed in TCGA.

### Quantitative real-time PCR assays (qRT-PCR)

qRT-PCR was used to test the relative expression of PSMB8 from BRAC cells. RNA was extracted by Trizol reagent and then exploited for the process of reverse transcription via a reverse transcription reagent kit (Toyobo, Osaka, Japan). Finally, qRT-PCR was conducted using THUNDERBIRD SYBR qPCR Mix (Toyobo, Osaka, Japan) with the machine of Applied Biosystems 7500. Each sample was conducted at least triplicate and the relative expression of PSMB8 was measured by the 2^−ΔΔCt^ calculation fomula compared to GAPDH expression. The sequences of the primers used for cDNA amplification were listed as followed: PSMB8 Forward: 5′-GCTGCCTTCAACATAACATCA-3′, and Reverse: 5′-CTGCCACCACCACCATTA -3′; GADPH Forward: 5′-GTCTCCTCTGACTTCAACAGCG-3′ and Reverse: 5′-ACCACCCTGTTGCTGTAGCCAA-3′.

### Epigenomic deconvolution

In the complicated background of tumor cell-type heterogeneity, we utilized the epigenomic deconvolution (EDec), a reference-free algorithm, to infer cell-type proportions from DNA methylation and clarify the immunocyte-specific expression pattern of PSMB8^15^. The simulations was based on an open source software EDec(http://genboree.org/theCommons/projects/edec) and the output file containing the mean and standard error estimates of cell type and subtype-specific gene expression profiles based on the TCGA BRCA dataset was directly utilized for the presentation(http://genboree.org/theCommons/documents/571).

### Statistical analysis

Gene expression data from the TCGA, GTEx, and CCLE databases were analyzed using Student’s t-test. The Kruskal Wallis test was used to evaluate PSMB8 expression in pan-cancer, and the Wilcoxon test was used to evaluate gene expression differences between normal and tumor tissues. OS was calculated using the Kaplan–Meier method, and survival curves were compared using log-rank tests. Pearson analysis was performed to evaluate the correlation between PSMB8 expression levels with checkpoint-related genes. All statistical analysis was conducted using R software (version 3.6.1). A *P*-value < 0.05 was considered statistically significant.

## Results

### Pan-cancer expression profile of PSMB8

**Figure 1:**
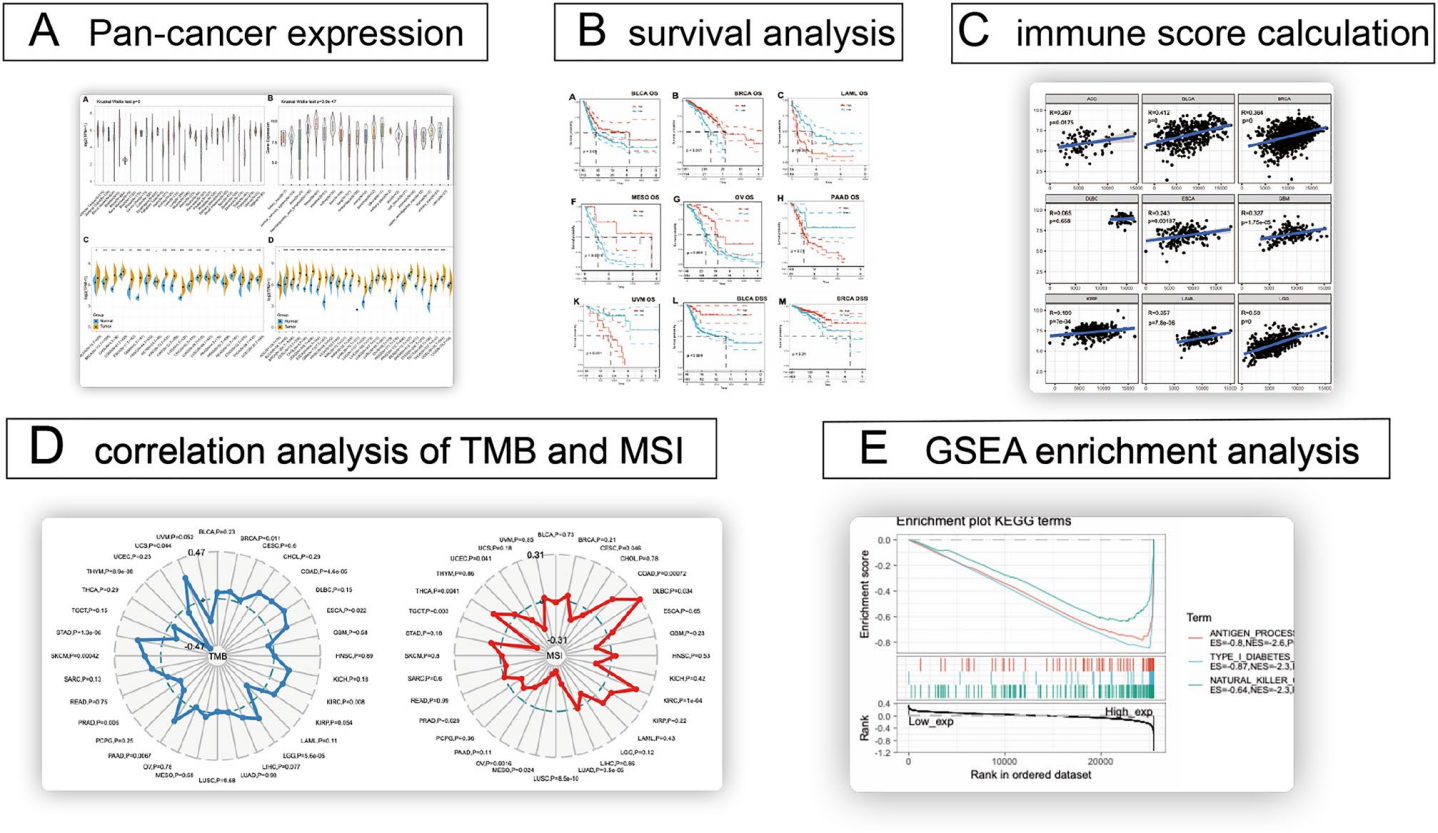
Flowchart illustrating the pan-cancer analysis.

**Figure 2:**
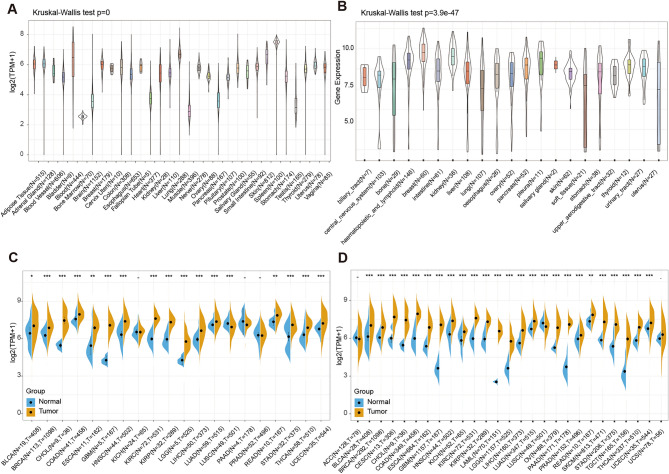
Expression of PSMB8 in TCGA cohorts, GTEx datasets, and CCLE cell lines. (**A**,**B**) It Significant variation of PSMB8 expression can be observed across different normal tissues and normal cell lines. (**C**,**D**) Differential expression between normal and tumor tissues in TCGA and GTEx databases. T, tumor; N, normal. X axis represented the number of tumor and normal samples. Y axis represented transcripts per million [log2(TPM + 1)]. *P < 0.05, **P < 0.01, and ***P < 0.001.

To start with, our research procedure towards PSMB8 in the pan-cancer was shown in Fig. 1. Previous studies investigating PSMB8 have alluded to two contradictory notions, one of which considered that elevated PSMB8 expression was adversely associated with tumor progression, such as in PTC, LAML, and GBM, while the other regarded PSMB8 as an immune-stimulatory factor with protective characteristics. Thus, we first performed a comprehensive evaluation of PSMB8 mRNA expression in normal tissues in the GTEx database and in cell lines in CCLE database. The results revealed that PSMB8 was expressed in relatively lower levels in the bone marrow, muscle, and testis, while expression was much higher in the spleen, bladder, and lung (Kruskal–Wallis test *P* < 0.05) (Fig. 2A). As shown in Fig. 2B, the PSMB8 expression in 22 normal cell lines extracted from the CCLE database was also observed with substantially significant differences (Kruskal–Wallis test: *P* = 3.9e−47).

Further, to distinguish between the two potential roles of PSMB8, we conducted a pan-cancer expression profile analysis using datasets from TCGA and the GTEx databases to compare differences in expression between tumor and normal tissue. The results obtained from the preliminary expression analysis of PSMB8 are presented in Fig. 2C,D. The analysis of differentially expressed PSMB8 in TCGA (Fig. 2C) revealed significant differences except for kidney chromophobe (KICH), pancreatic adenocarcinoma (PAAD), and prostate adenocarcinoma (PRAD). Subsequently, we combined gene expression from normal samples in the GTEx with those from TCGA and generated a new plot. As shown in Fig. 2D, PSMB8 was overexpressed in tumor tissues compared to normal tissues on average, except for lung squamous cell carcinoma (LUSC) and adrenocortical carcinoma.

### Prognostic significance of PSMB8 across cancers

**Figure 3:**
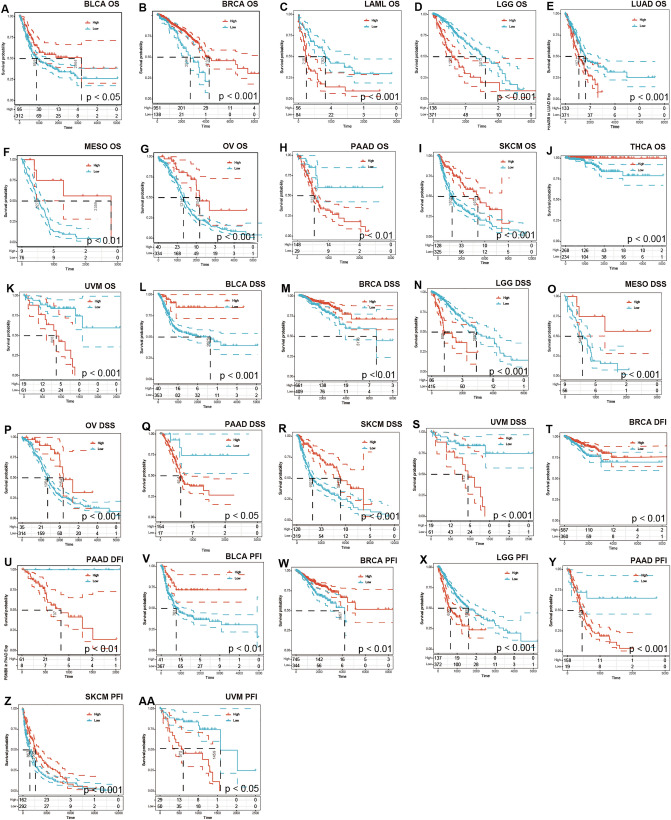
Kaplan–Meier survival curves of survival comparing high and low expression of PSMB8 in pan-cancer derived from TCGA. (**A**–**K**) Overall survival differences between groups in BLCA, BRCA, LAML, LGG, LUAD, MESO, OV PAAD, SKCM, THCA, and UVM. (**L**–**S**) Disease-specific survival differences between groups in BLCA, BRCA, LGG, MESO, OV, PAAD, SKCM, and UVM. (**T**–**U**) Disease-free interval difference between groups in BRCA and PAAD. (**V**–**AA**) Progression-free interval differences between groups in BLCA, BRCA, LGG, PAAD, SKCM and UVM.

The first part of our analysis explored the expression profile of PSMB8, which raised the issue of its prognostic value. We utilized TCGA database comprising Affymetrix microarray data of 33 cancer types as well as their survival-related follow-up to investigate the impact of PSMB8 expression on OS, DSS, DFI, and PFI. Cox regression analysis revealed that PSMB8 denoted favorable OS and DSS in bladder urothelial carcinoma (BLCA), breast invasive carcinoma (BRCA), ovarian serous cyst adenocarcinoma, mesothelioma (MESO), and skin cutaneous melanoma (SKCM). Our results regarding OS, DSS, PFI showed a robust carcinogenic-predictive role of PSMB8 in brain lower grade glioma (LGG), uveal melanoma (UVM), and PAAD. Furthermore, high expression of PSMB8 was associated with poor survival in LAML and lung adenocarcinoma (LUAD). With regard to DFI, increased PSMB8 expression showed a satisfactory prognosis in BRCA patients, and a poor outcome in PAAD, instead. The significant results of the survival analysis are plotted in Fig. 3. All results of all cancer types at all survival times was presented in 'Supplementary Data S1'.

### Correlation of PSMB8 expression with immune infiltration level and components of immune cells

Previously, a variety of studies have revealed that TIICs influence the response rate of immunotherapy, the efficacy of chemotherapy, and the ultimate prognosis of malignancies. Chen et al. demonstrated the seven steps of the cancer-immunity cycle, which have become the basic framework of cancer immunotherapy research. Portrayed as one of the constitutive proteasome genes (CP), expression of PSMB8 has been associated with levels of MHC-I, the antigen-presenting cells, and TIILs, and consequently plays a crucial part in the above-mentioned immunity cycle^16^. Albeit proteasome inhibitors, such as bortezomib, may induce proteotoxic stress and apoptotic activity as targeted therapy, there are currently investigative areas worthy of attention involving the suppression of immunoproteasome (i-proteasome) activity to induce immune evasion and metastatic progression^17^. Therefore, it is necessary to evaluate the immune properties of PSMB8 in the context of pan-cancer.

The online TIMER database was exploited to calculate the expression of the immune cells in association with a target gene. We found that all six subtypes of immune infiltration cells were associated with elevated PSMB8 expression in the following tissues: kidney renal papillary cell carcinoma (KIRP), brain lower grade glioma (LGG), liver hepatocellular carcinoma (LIHC), lung adenocarcinoma (LUAD), lung squamous cell carcinoma (LUSC), prostate adenocarcinoma (PRAD), sarcoma (SARC), skin cutaneous melanoma (SKCM), stomach adenocarcinoma (STAD), and testicular Germ Cell Tumors (TGCT). In contrast, there seemed to be no immune infiltration-associated expression patterns with PSMB8 in cholangiocarcinoma (CHOL) and rectum adenocarcinoma (READ). Furthermore, the Spearman correlation coefficient determined for the immune infiltration levels of B cells, CD8+ T cells, CD4+ T cells, macrophages, neutrophils, and dendritic cells were notably significant in 23, 23, 25, 23, 19, and 26 cancer types, which comprised a large proportion of the 32 cancer types interrogated (Supplementary Figures S1–S8).

**Figure 4:**
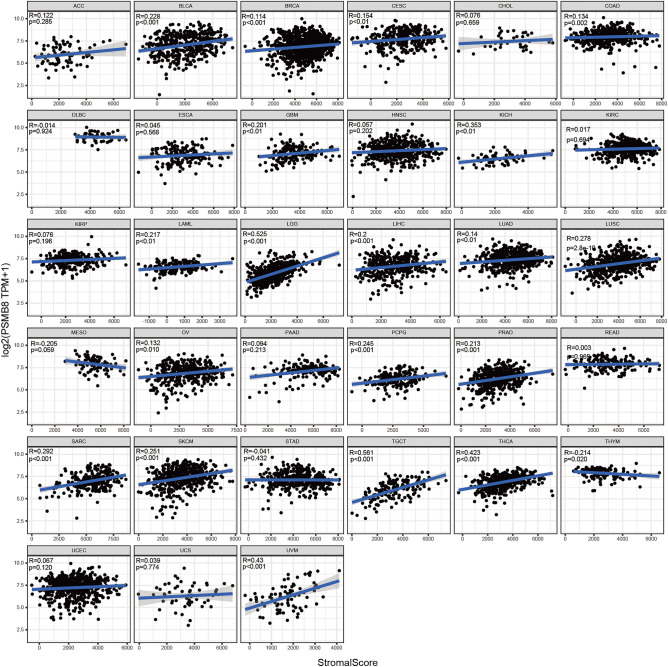
Correlation analysis between PSMB8 expression in 33 types of cancers and stromal score.

**Figure 5:**
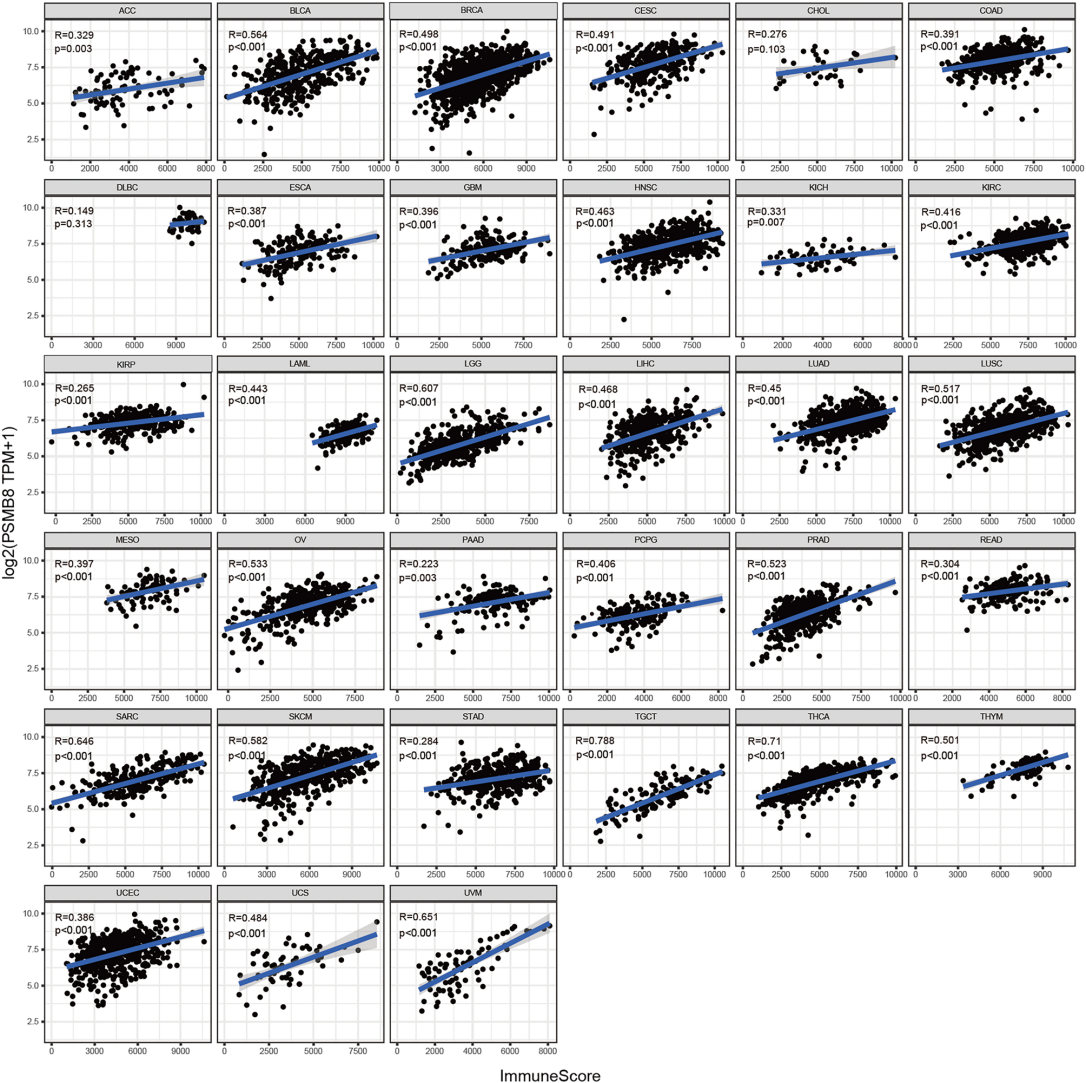
Correlation analysis between PSMB8 expression in 33 types of cancers and immune score.

**Figure 6:**
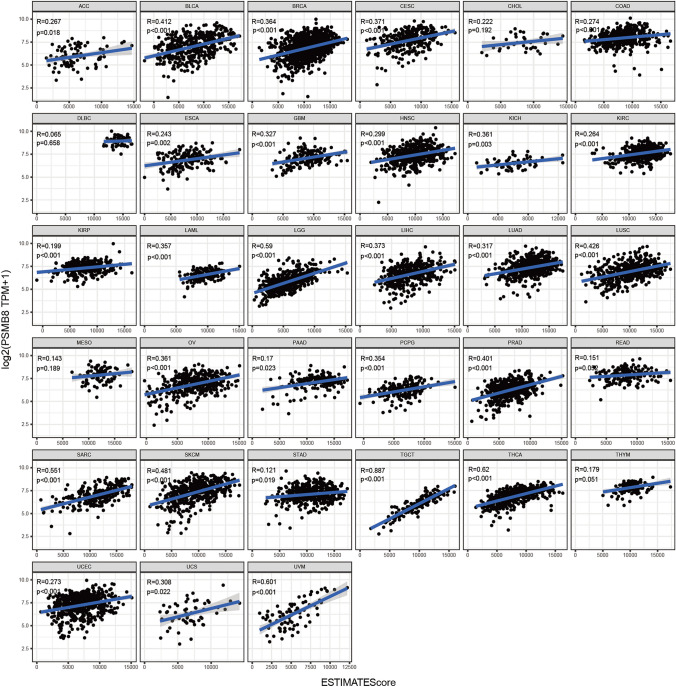
Correlation analysis between PSMB8 expression in 33 types of cancers and the estimated immune score.

An additional immune algorithm, called the ESTIMATE algorithm, was applied. Three of the calculated scores were the StromalScore, ImmuneScore, and ESTIMATEScore, which were determined to be directly proportional to the ratio of the corresponding stromal, immune components, and their aggregates. In most cancer types, PSMB8 emerged as a statistically significant marker of the TME status. Thus, correlations between PSMB8 expression and StromalScore, ImmuneScore, and ESTIMATEScore were determined in 20, 31, and 29 cancer types, respectively (Figs. 4, 5, 6). The top three tumors most significantly associated with overexpression of PSMB8 based on the ESTIMATEScore were listed the following: TGCT, THCA, and UVM. Nevertheless, CHOL and DLBC showed no statistically significant association across all three scores. The ESTIMATE outcome generated interesting insight into THCA, which ranked second in the ESTIMATEScore as well as the ImmuneScore, and ranked fourth in the StromalScore, and thus, hereinafter we switched our focus to the role of PSMB8 in THCA.

To determine the underlying mechanism of PSMB8 in the enhanced immunological response, we calculated the Spearman correlations of PSMB8 expression with immune-stimulatory and immunoinhibitory factors. We set the statistical criteria as *P* < 0.05. As illustrated in the heatmap, the bulk of cancer types were positively associated with most immune elements, with the exception of CHOL, DLBC, ESCA, GBM, KICH, MESO, and READ. These seven cancer types identified a subset in which the TME was irrelevant to PSMB8 expression, and thus the predictive role of PSMB8 was far from optimal in this subgroup, which is frequently encountered in the search for tumor biomarkers.

**Figure 7:**
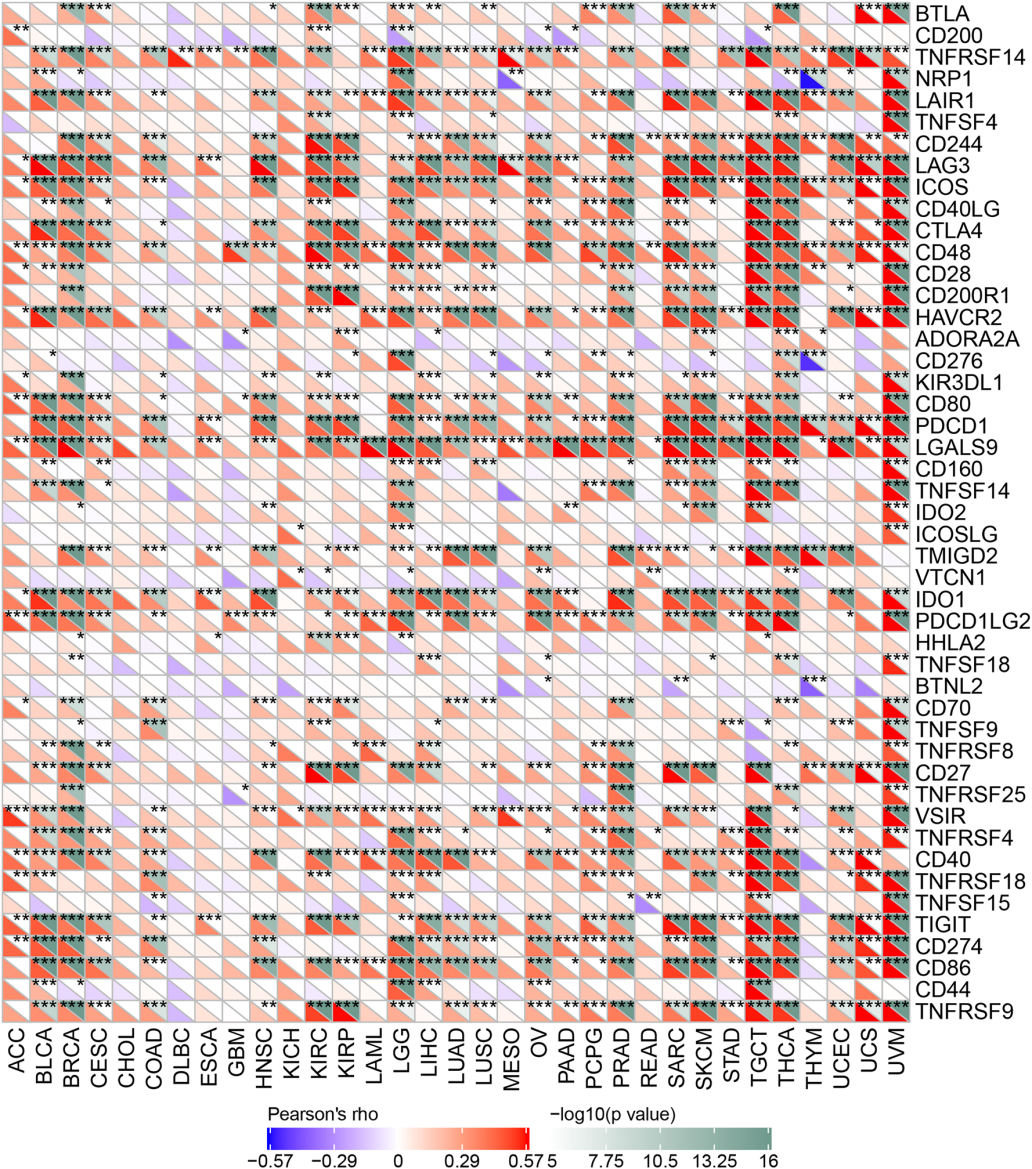
Each cancer was conducted the correlation analysis between 47 immune checkpoint genes and the gene expression of PSMB8, which was presented in the heatmap. The spearman correlation coefficients were filled in accordance with the corresponding colors at the bottom of chart (*P < 0.05, **P < 0.01, and *** *P* < 0.001).

**Figure 8:**
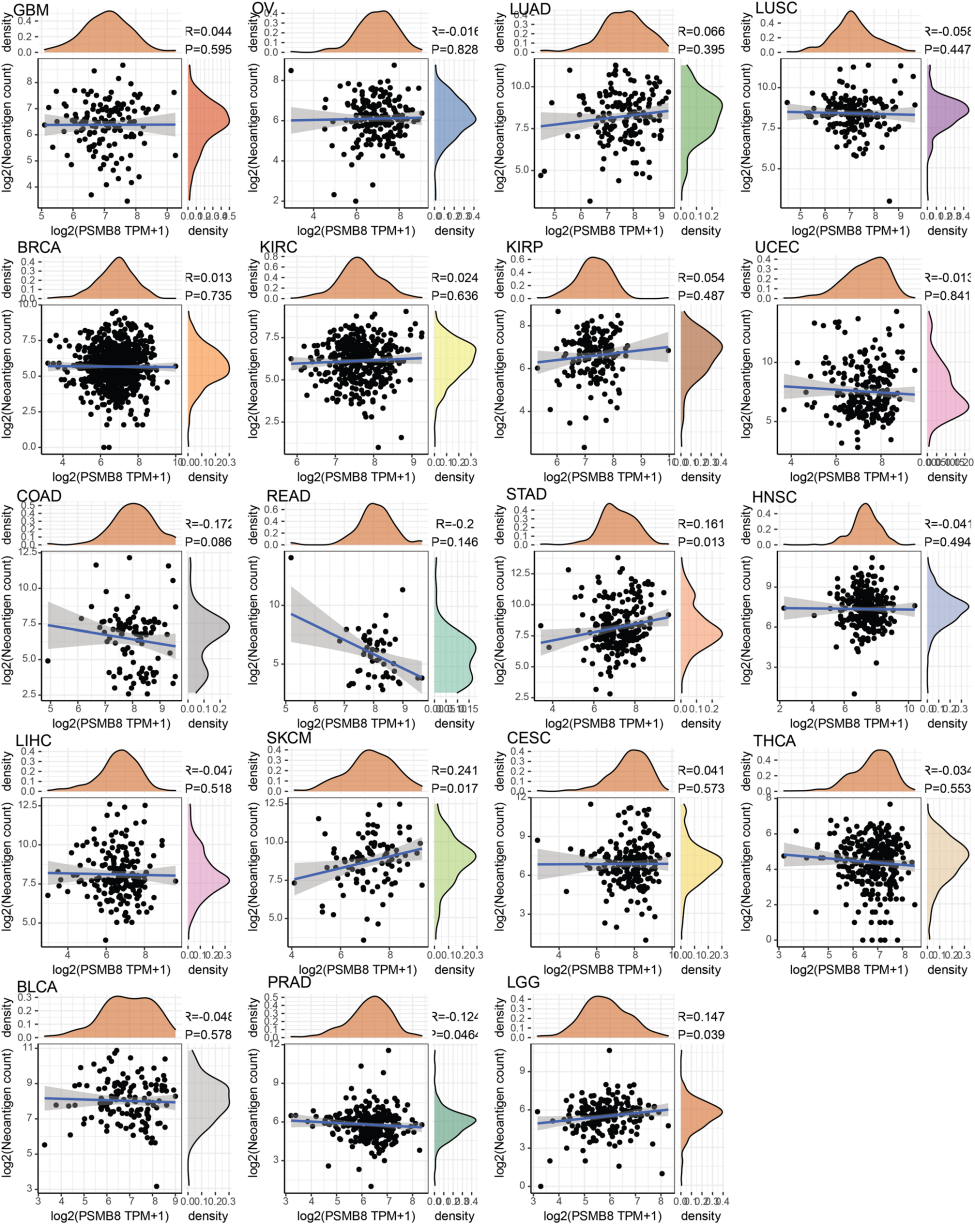
Correlation analysis between the level of neoantigens and PSMB8 expression in pan-cancer.

Our results revealed that a positive relationship between PSMB8 expression and the levels of immune checkpoint genes in various tumors, such as TGCT, THCA and UVM (Fig. 7). Thus, these findings indicated that PSMB8 could play a role in tumor immunity by regulating the expression of these immune checkpoint genes. As shown in Fig. 8, we determined that the expression of PSMB8 was positively correlated with the number of neoantigens only in SKCM tumor tissues (R = 0.241, *P* < 0.05). Of note only a *P-*value of < 0.05 and R > 0.20 may be considered as significant and positive, respectively.

**Figure 9:**
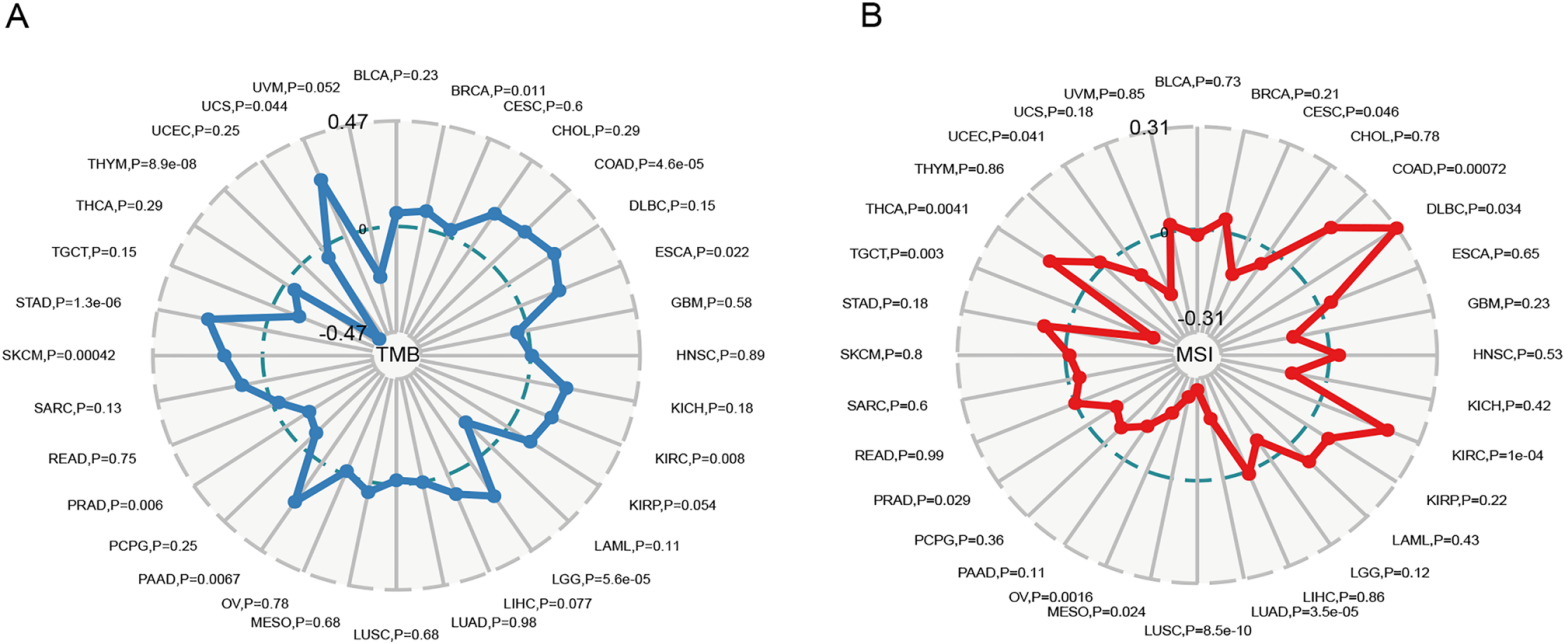
Correlation of PSMB8 expression with tumor mutation burden (TMB) and microsatellite instability (MSI) in multiple cancer types. (**A**) Correlation between TMB and PSMB8 expression. (**B**) Correlation between MSI and PSMB8 expression. Spearman’s correlation coefficients are shown above the bar graphs. (Spearman Correlation test, *P* < 0.05 was considered statistically significant).

The TMB and MSI have conceptually emerged as predictors of an effective immune response, which are promoted within the spectrum of tumor exacerbation. We further evaluated the strength of the relationship between PSMB8 expression and the TMB or MSI in pan-cancer. The most distinct correlation coefficients in the analysis of the TMB and MSI were ± 0.47 and ± 0.31, respectively. In detail, a significant correlation was identified between overexpression of PSMB8 and an increased TMB in UCS, STAD, SKCM, PAAD, LGG, KIRC, ESCA, COAD, and BRCA tissues, while an opposite association was observed in THYM and PRAD tumor tissues (Fig. 9A). Similarly, increased PSMB8 expression was significantly associated with an increased MSI in THCA, KIRC, DLBC, and COAD tissues and was inversely correlated with in UCEC, TGCT, PRAD, OV, MESO, LUSC, LUAD, and CESC (Fig. 9B).

### Association analysis of PSMB8 with DNA mismatch repair genes and methyltransferases

**Figure 10:**
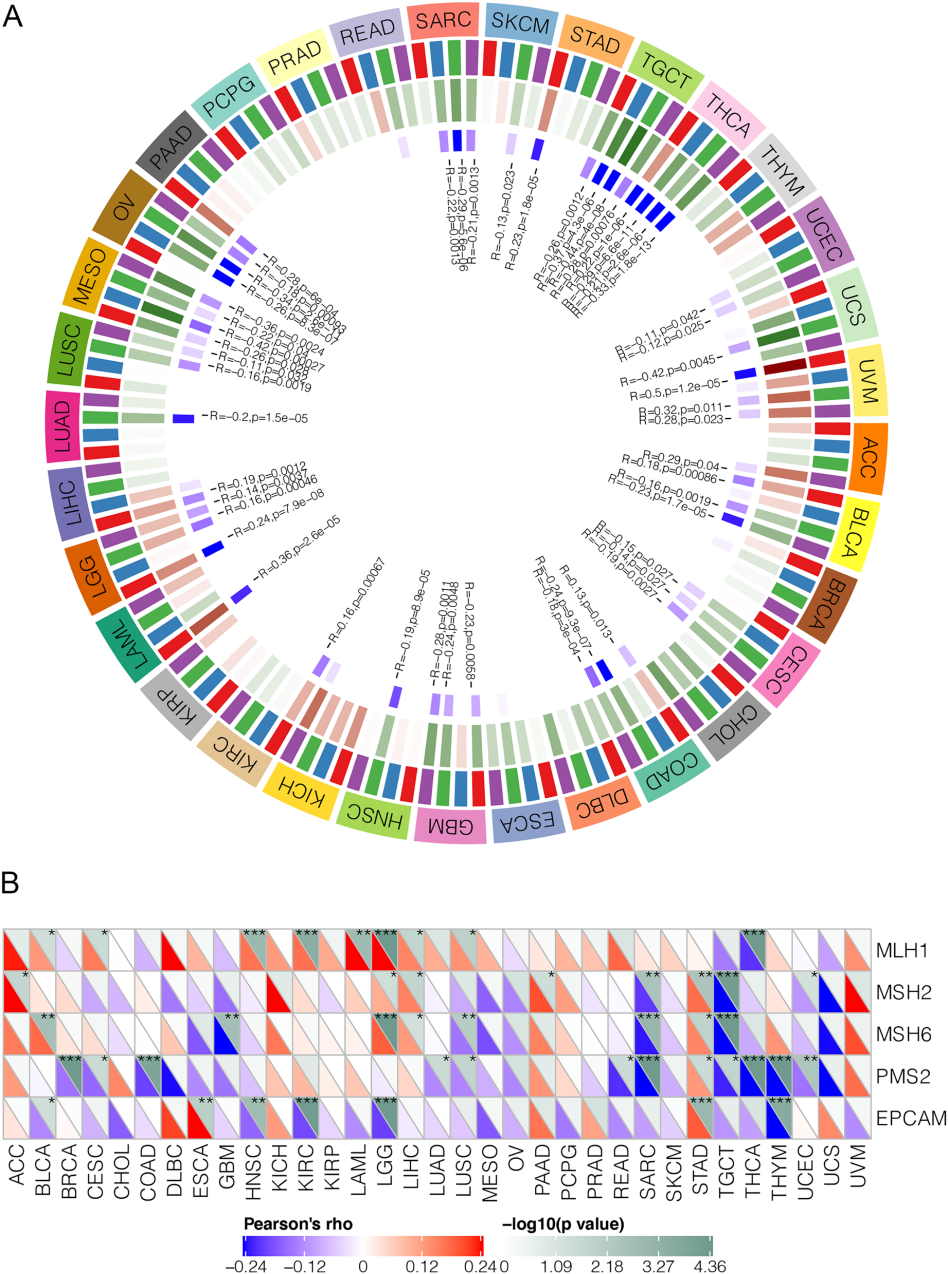
Correlation analysis between PSMB8 expression and (**A**) four methyltransferases and (B) five MMR genes in various tumor types. The color of the second outer ring represented the orderly represented Dna methyltransferases (Dnmt1, Dnmt2, Dnmt3 and Dnmt4). The Pearson’s rho and − log10(p value) was presented below.

As shown in Fig. 10A, a significant correlation between PSMB8 and four methyltransferase expression levels could be observed in most cancer types. In addition, DNA mismatch repair gene expressions were almost inversely correlated with the PSMB8 expression in pan-cancer, whereas MLH1 was of positive correlation (Fig. 10B). Our analysis revealed that PSMB8 could regulate epigenetic status in pan-cancer.

### Gene-annotation and pathway enrichment analysis

**Figure 11:**
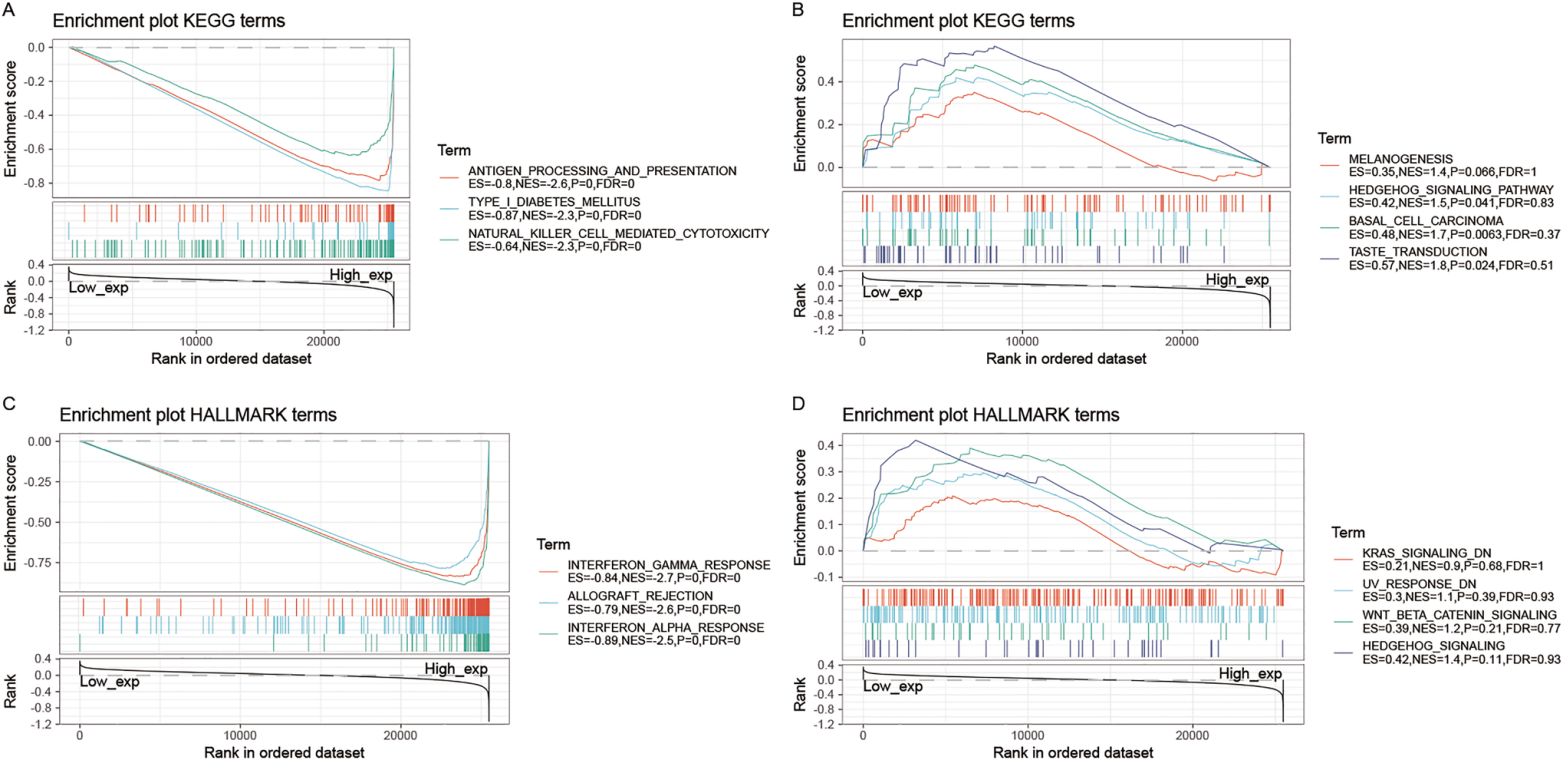
GSEA for samples with high PSMB8 expression and low expression. (**A**) The enriched gene sets in following KEGG analysis using high PSMB8 expression samples. (**B**) The enriched gene sets in KEGG by samples with low PSMB8 expression. (**C**) Enriched gene sets in HALLMARK collection, the immunologic gene sets, by samples of high PSMB8 expression. (**D**) Enriched gene sets in HALLMARK by the low PSMB8 expression. Each line represents one gene set with a unique color, and up-regulated genes located in the left approaching the origin of the coordinates, by contrast the down-regulated genes are indicated on the right of the x-axis. Only gene sets with NOM *P* < 0.05 and FDR *q*-value < 0.05 were considered significant and displayed in the plot.

To uncover the potential signaling pathways and immunocompetences associated with the involvement of PSMB8 in tumorigenesis, we applied GSEA using KEGG and HALLMARK terms. Only enriched gene sets with the absolute value of normalized enrichment score (NES) > 1, nominal (NOM) *P* < 0.05, and an FDR q-value < 0.25 were considered statistically significant. As shown in Fig. 11A,C, KEGG and HALLMARK enriched terms showed that overexpression of PSMB8 was mainly associated with immunological processes, including antigen processing and presentation (NES = − 2.6, NOM *P* < 0.05), natural killer cell-mediated cytotoxicity (NES = − 2.3, NOM *P* < 0.05), allograft rejection (NES = − 2.6, NOM *P* < 0.05), the interferon (IFN)-gamma response (NES = − 2.7, NOM *P* < 0.05), and the IFN-alpha response (NES = − 2.5, NOM *P* < 0.05). Furthermore, high expression of PSMB8 was associated with metabolic syndromes, such as type I diabetes mellitus (NES = − 2.3, NOM *P* < 0.05). However, there was no significant enrichment in the analysis based on low expression of PSMB8 (Fig. 11B,D).

### Native validation of PSMB8 expression in the BRCA subtypes

**Figure 12:**
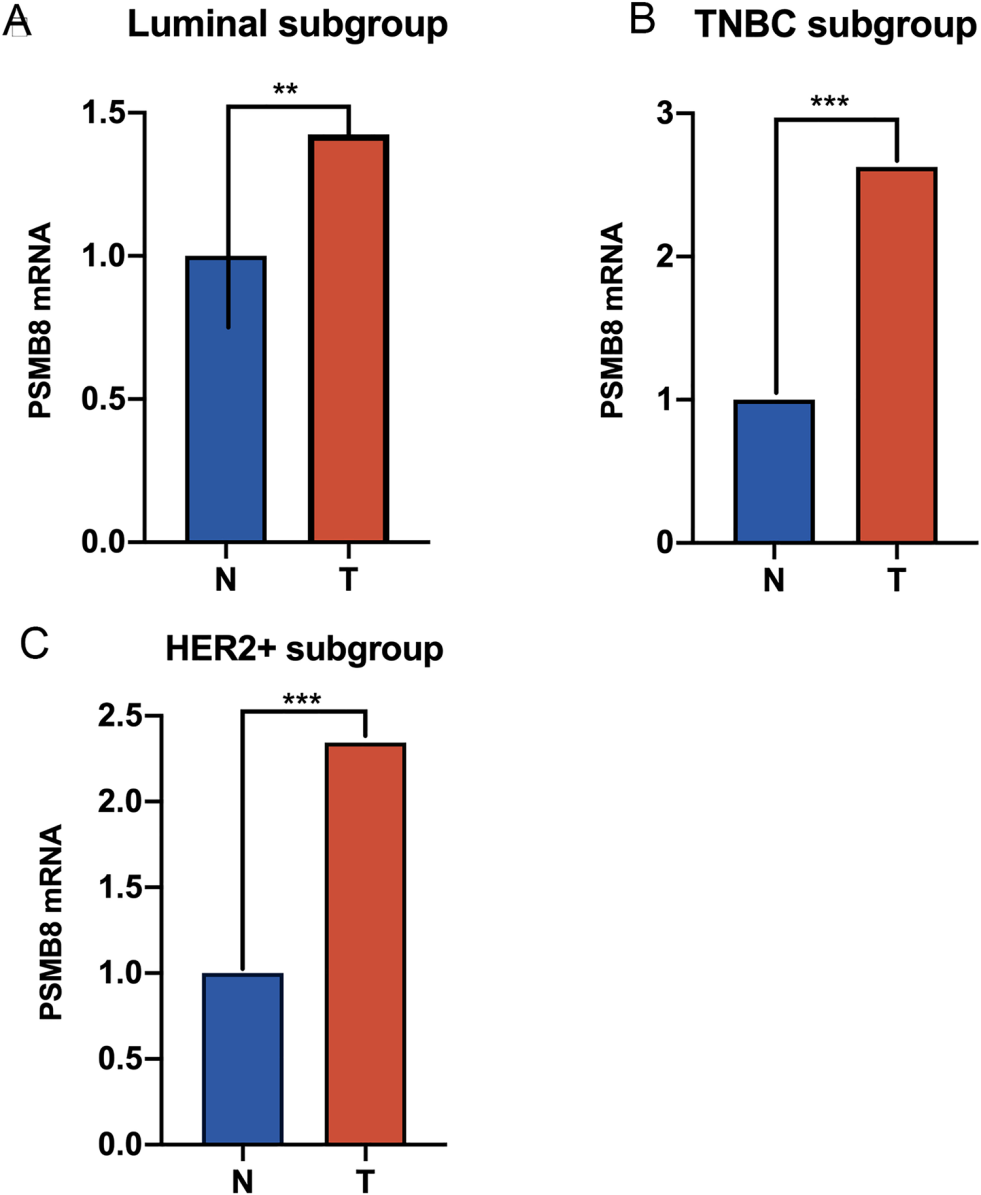
PSMB8 is overexpressed compared to adjacent normal tissues in local BRCA tissues by Student's t-test, which is composed of (**A**) luminal subtype, (**B**) TNBC subtype, and (**C**) HER2+ subtype. 2^−ΔΔCt^ is used to present the fold change in qRT-PCR experiment. (*P < 0.05, **P < 0.01, and ***P < 0.001).

To further validation, we chose 60 pairs BRCA tissues and their corresponding adjacent normal tissues to perform qRT-PCR. Our local specimens were all sampled from the operation room in the First Affiliated Hospital of Wenzhou Medical University, which were made up of luminal (HR+, HER2−), TNBC (HR−, HER2−), and HER2+ (HR−, HER2+) subgroups. And Fig. 12 directly show the disparity tendency of PSMB8 expression between tumor and normal tissues with significantly statistical difference (*P < 0.05, **P < 0.01, and ***P < 0.001).

### Immunocyte-specific expression pattern of psmb8 in brca by epigenomic deconvolution

**Figure 13:**
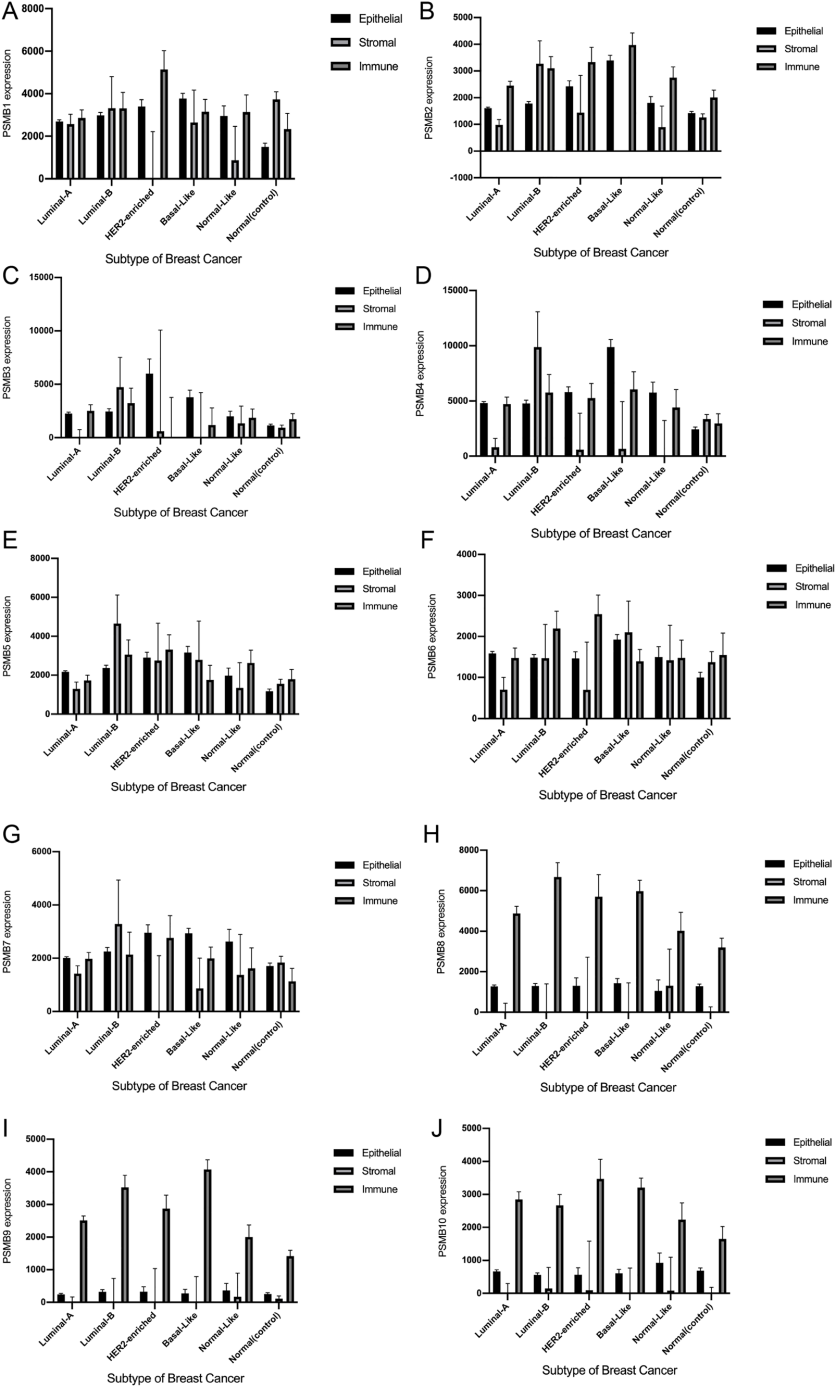
Cell type-based expression profile derived from TCGA BRCA in 10 catalytic proteasome subunits. And the immunoproteasome subunit, comprising (H)PSMB8, (I)PSMB9 and (J)PSMB10, was observed in an immunocyte-specific expression pattern.

Tumor heterogeneity partially reflected in the proportion of the various cell-types in a tumor (composed of immunocyte, stromal cell, tumor cell and normal cell), aroused the desire to micro dissect and then infer cell-type composition. By applying EDec to TCGA BRCA queue, the expression profile was delineated precisely based on the subtype and cell type. The mean and standard error estimates of immunoproteasome subunit encoding gene expression was filed in Supplementary Data S2. And the comparative histogram revealing the expression pattern of 10 catalytic subunit validated the immunocyte-specific profile of PSMB8 (shown in Fig. 13).

## Discussion

The awareness of remodeling an efficient anti-tumor TME improved, along with the widely-spread application of immune checkpoint blockade tumor therapy. With the exception of immunologically-hot cancer types benefiting much from blocking immune checkpoints, the overall objective response rates of other tumors merely ranged from 15 to 25%, which was far from satisfactory^18^. Recently, the third generation of combined immunotherapy strategies, integrating an immunomodifier with immune checkpoint inhibition (ICI), has achieved synergistic effects in the maintenance of a dominant and enhanced immunological response^19^, which fully clarified a positive anti-tumor immune modification was of significance when ICI didn't work.

PSMB8, a catalytic subunit of immunoproteasomes, plays a critical role in the process of proteolysis to generate the antigenic epitopes, which are in turn transmitted to MHC class I molecules for further antigen-presentation. Thus, the nature of antigen presentation, to some extent, significantly enhanced the pool of MHC-I compatible peptides. The increased release of IFN-gamma by the TIIL in the TME trigger the incorporation of immuno-subunits into the catalytic core of the proteasome and transform the excessive proteasomes into immunoproteasomes. The IFN-gamma inducible genes (PSMB8/β5i, PSMB9/β1i, PSMB10/β2i), together with their chaperones have command of the tumor vulnerability to antigen-dependent killer cells^20^. Thus, essential subunits (more explicitly referred to PSMB units) were fundamentally engaged in the immunoproteasome transformation, acting against the construction of anti-tumor immunity.

On the other hand, malignancies are usually subjected to proteotoxic stress, under which circumstances tumorigenic proteins induced by genomic aberrations are assembled at the expense of proteasome-promoted activities regulating proteostasis^21^. Accordingly, a variety of tumors addictively resort to immunoproteasome activities. An experimental study determined that down-regulated incorporation of PSMB8 into immunoproteasome could attenuate its formation, laying the foundation of the key part of PSMB8 in the proteolytic activity and protein homeostasis in maintenance of tumor development. In a previous study, the immunoproteasome (β1i-β2i-β5i) and the two intermediate proteasomes (β1-β2-β5i and β1i-β2-β5i), except for the standard proteasome (β1-β2-β5), exhibited the comparative efficiency of degrading ubiquitinated or oxidized proteins, indicating the more significant role of β5i/PSMB8 than β1i/PSMB9 and β2i/PSMB10.

The above contradictory arguments underlined a chaotic picture of the functional orientation of PSMB8 in neoplasms. To sum up, PSMB8, the intrinsic APG, capable of enlarging the pool of MHC-I compatible peptides, credibly reinforced the capacity of antigen presentation to the immune system and in turn antigen-dependent cytotoxicity. On the reverse side, more in-depth mechanism studies discovered its interaction with oncogenic signaling pathway and the essential function in oncoprotein homeostasis.

As far as we are concerned, in consideration of the immunological relevance, it would be better to analyze the candidate gene, PSMB8, in the circumstance of specific TIME. Szekely et al. noticed a coordinated down regulation of PSMB8-10(β5i, β1i, β2i) in the metastases than the primary breast tumor, together with the TIL counts and immunomodulator signatures. We boldly speculate that PSMB8 alone could not make sense to the prognosis of immunologically-inert metastases and the other cancer types likewise. And just like the circumstantial impression made by PD-(L)1, only in the immune activation background, could the antigen-presentation effect be stimulated sufficiently; Otherwise the PSMB8 orientate its function towards the ontogenetic downstream signaling, such as PSMB8-mediated PI3K/PTEN/AKT /MTOR pathway activation, which was verified to facilitate tumorigenesis.

This gene has not yet been fully exploited, particularly in the immuno-regulation level. Hence, our study which investigated the general applicability of the antigen-presentation regulator PSMB8 as a prognostic biomarker in pan-cancer from an immuno-oncological perspective, could provide a rational and theoretical foundation for future mechanism studies.

As high-throughput sequencing techniques become relatively easier to perform, large-scale public databases have flourished. Independent datasets with pan-cancer expression profiles were acquired from TCGA, GTEx, and CCLE, which include normal tissues, tumor tissues, and cancer cell lines. We extracted the expression of PSMB8 and visually rendered the comparison in the pan-cancer. Using GTEx and TCGA, we found that PSMB8 expression was generally lower in normal tissues, while exhibited varied expression in tumor cell lines of CCLE. Next, we explored differences in expression between tumor and normal tissues using TCGA datasets and determined that PSMB8 was generally prone to be over-expressed in the most tumor tissues compared to normal tissues, except for LUSC, PAAD, PRAD, and KICH tumors. When data from normal tissues in the GTEx was added to the TCGA, PSMB8 expression was higher in PAAD, PRAD, and KICH than adjacent normal tissues. Thus, only PSMB8 expression in LUSC was inconsistent and finally drew a conclusion that overexpression of PSMB8 appeared more frequently in the tumor tissues than in their normal counterparts.

We next attempted to identify the prognosis-predictor value for PSMB8. Our findings showed that high expression of PSMB8 in BLCA, BRCA, MESO, OV, and SKCM was successful in predicting better OS and DSS. In view of these survival outcomes, the function of PSMB8 was orientated more like a protective role. Conversely, increased PSMB8 expression revealed poor overall survival for LAML, LGG, LUAD, PAAD, and UVM based on Kaplan–Meier analysis. In a word, our results sustained coherence with prior studies, as well as revealed the functional pleiotropy of PSMB8, albeit with unknown molecular mechanisms.

Given the overpowering energy of the immune microenvironment implicated in the process of tumorigenesis, a significant correlation-analysis in pan-cancer was conducted. More than two-thirds of cancer types exhibited a significant association between PSMB8 and the immune infiltrating cells. Since the TME are constantly in a state of flux, tumor progression along a relatively unmanageable pathway may demand a metabolic reprogramming obtained from the TIICs, which leads to subsequent vicious circle of tumor differentiation. Unfortunately, CHOL was considered an exception on account of the irrelevance between PSMB8 and the TME constituents, including both immune and stromal elements, which could be partly in explanation of its very poor survival rates.

Proteasomes promoted the degradation of endogenous and exogenous antigens presented by the MHC class I system, and thus were linked to antigen presentation. The maturation of dendritic cells (DC), which are acknowledged as the most powerful APCs, orchestrate the changes in proteasomal composition. More immunoproteasomes populate DCs, and inducible cytotoxic T lymphocytes (CTLs) are unable to properly identify tumor cells that constitutively express these proteasomes^22^. Inhibition of the catalytic subunit involved in immunoproteasome-transformed activities, herein referred to as PSMB8, promoted an alteration of antigenic peptide-repertoires expressed by antigen-loaded DCs. In our ESTIMATE algorithm-based analysis, all cancers types except for READ, CHOL, UVM, THYM, ESCA, GBM, and DLBC, presented a significant correlation between PSMB8 expression and DC. In brief, DCs were the most closely-associated with PSMB8 levels in the scope of pan-cancer, and both were observed to correlate with the antigen-presenting system.

Notably, one of the strongest positive associations was observed between PSMB8 expression in BRCA and immune markers, which comprised the Estimate Score, various levels of TIIC, TMB, and MSI. Validation of the PSMB8 in TCGA BRCA cohort through EDec algorithm, supported its immunological relationship.

To sum up, as there have not been any credible immuno-oncology evidences emerging in the previous decades, we explored this antigen presentation gene (APG) from an immunological standpoint. As justified by previous experimental or bioinformatic studies combined with our pan-cancer survey described herein, different tumor types and their corresponding microenvironments did not alter the essential antigen-presentation function of PSMB8, but definitely bent its orientation concerning tumor prognosis through the downstream oncogenic signaling. Szekely et al. noticed a coordinated down regulation of PSMB8-10 (β5i, β1i, β2i) in the metastases than the primary breast tumor, together with the TIL counts and immunomodulator signatures. We boldly speculate that PSMB8 alone could not make sense to the prognosis of immunologically-inert metastases and the other cancer types likewise. And just like the circumstantial impression made by PD-(L)1, only in the immune activation background, could the antigen-presentation effect be stimulated sufficiently.

Nevertheless, this study extending across multiple databases presented some limitations. First, we conducted a bioinformatic analysis of PSMB8, from which it was difficult to assess the value of clinical transformation. Although it was clear that more mechanism analysis added to our preliminary analysis would be useful to understand the details of interaction and the role of PSMB8 as an immunomodulator.

In addition, since the resources in all the databases were tissue-derived, these findings cannot be validated with in vitro/in vivo models. Besides, an alternative technique to qPCR, such as RNAseq/microarray would be more precise. In the comparative biodiversity study, the methodological factors, such as preservation buffer, template concentration, DNA polymerase, PCR enhancer, was indeed identified to introduce the critical variability.

Finally, the polytrophic function of PSMB8 may represent an initial effect, and the underlying mechanisms engaged in tumor activities remain still elusive. Our pan-cancer research merely embodied an immune-related pan-cancer analysis and awaits future investigation into the mechanism involved in tumorigenesis.

## Supplementary Information

Supplementary Information 1.

Supplementary Information 2.

Supplementary Information 3.

## Data Availability

The data sets and additional images supporting the conclusions of this study are included in this article. Raw data are available on the main electronic data storage system of the First Affiliated Hospital of Wenzhou Medical University, and access can be provided upon request to the authors. Ethical approval for this study was obtained from the Ethics Committee of the First Affiliated Hospital of Wenzhou Medical University.

## Abbreviations

APCs: Antigen-presenting cells
APG: Antigen-presenting gene
ACC: Adrenocortical carcinoma
BLCA: Bladder urothelial carcinoma
BRCA: Breast invasive carcinoma; ovarian serous cystadenocarcinoma
CCLE: The Broad Institute Cancer Cell Line Encyclopedia
CESC: Cervical squamous cell carcinoma and endocervical adenocarcinoma
CHOL: Cholangiocarcinoma
CI: Confidence intervals
COAD: Colon adenocarcinoma
CTLs: Cytotoxic T lymphocytes
DCs: Dendritic cells
DFI: Disease-free interval
DLBC: Lymphoid neoplasm diffuse large B-cell Lymphoma
DSS: Disease-specific survival
ESCA: Esophageal carcinoma
GBM: Glioblastoma multiforme
GSEA: Gene Set Enrichment Analysis
GTEx: The Genotype-Tissue Expression
HNSC: Head and neck squamous cell carcinoma
HRs: Hazard ratios
KICH: Kidney chromophobe;
KIRC: Kidney renal clear cell carcinoma
KIRP: Kidney renal papillary cell carcinoma
LAML: Acute myeloid leukemia
LGG: Lower grade glioma
LUAD: Lung adenocarcinoma
LUSC: Lung squamous cell carcinoma
MESO: Mesothelioma
MHC: Major Histocompatibility Complex
MSI: Microsatellite instability
NES: Net enrichment score
NIK/NF-kB: Nuclear factor kappa-light-chain-enhancer of activated B cells;
OS: Overall survival
OV: Ovarian serous cystadenocarcinoma
PAAD: Pancreatic adenocarcinoma
PCPG: Pheochromocytoma and paraganglioma
PFI: Progression-free interval
PRAD: Prostate adenocarcinoma
READ: Rectum adenocarcinoma
SARC: Sarcoma
SKCM: Skin cutaneous melanoma
STAD: Stomach adenocarcinoma
TCGA: The Cancer Genome Atlas
TGCT: Testicular Germ Cell Tumors
THCA: Thyroid carcinoma
THYM: Thymoma
TIC: Tumor-infiltrating immune cell
TMB: Tumor mutation burden
TME: Tumor microenvironment
UCEC: Uterine corpus endometrial carcinoma
UCS: Uterine carcinosarcoma
UVM: Uveal melanoma
