# Supplementary material for: Pan-cancer analysis of the prognostic and immunological role of PSMB8
Source: Sci Rep. 2021 Oct 14;11:20492. doi: 10.1038/s41598-021-99724-9 (PMC8516870; doi:10.1038/s41598-021-99724-9)

Supplementary Material

Supplementary Data S1: Kaplan–Meier survival outcomes in pan-cancer derived from TCGA, including the hazard ratios as well as its p-values.

Supplementary Data S2: The mean and standard error estimates of the expression concerning eleven catalytic proteasome subunit encoding genes.

Supplementary Figure S1-8

Supplementary Figure S1: Correlation between six immune cell inﬁltration scores and PSMB8 mRNA expression in ACC, BLCA, CESC, and CHOL.


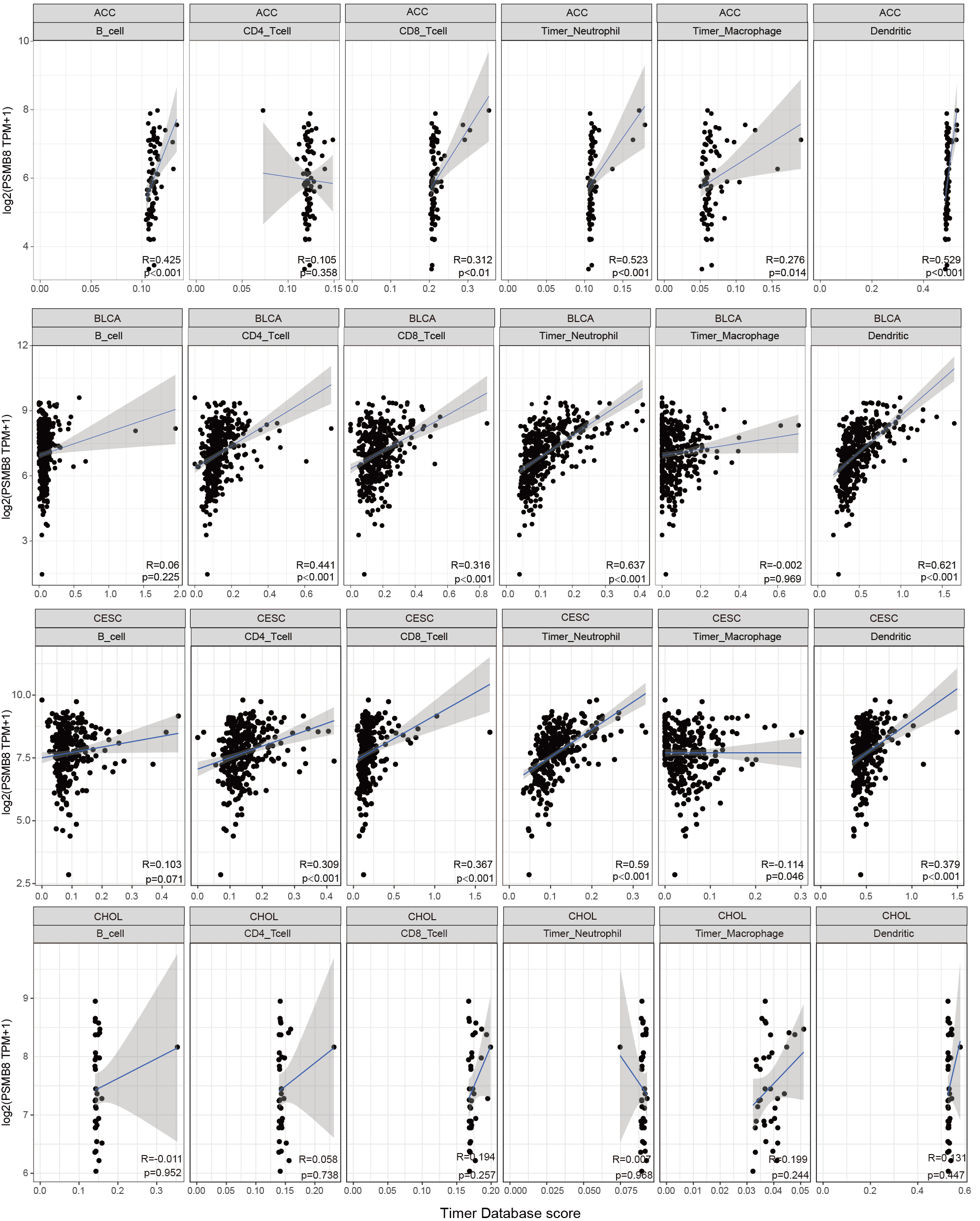


Supplementary Figure S2: Correlation between six immune cell inﬁltration scores and PSMB8 mRNA expression in COAD, DLBC, ESCA, and GBM.


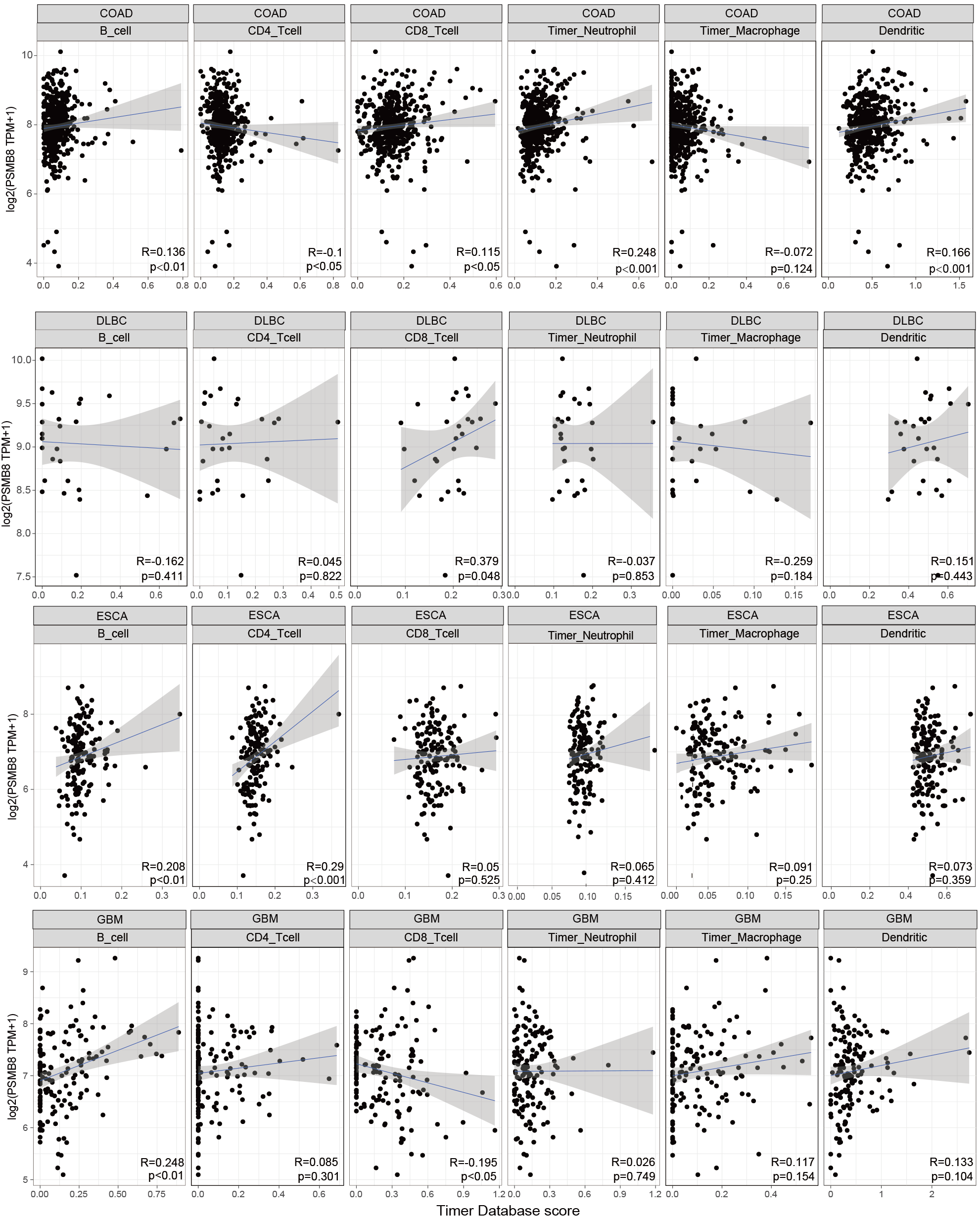


Supplementary Figure S3: Correlation between six immune cell inﬁltration scores and PSMB8 mRNA expression in BRCA, HNSC, KICH, and KIRC.


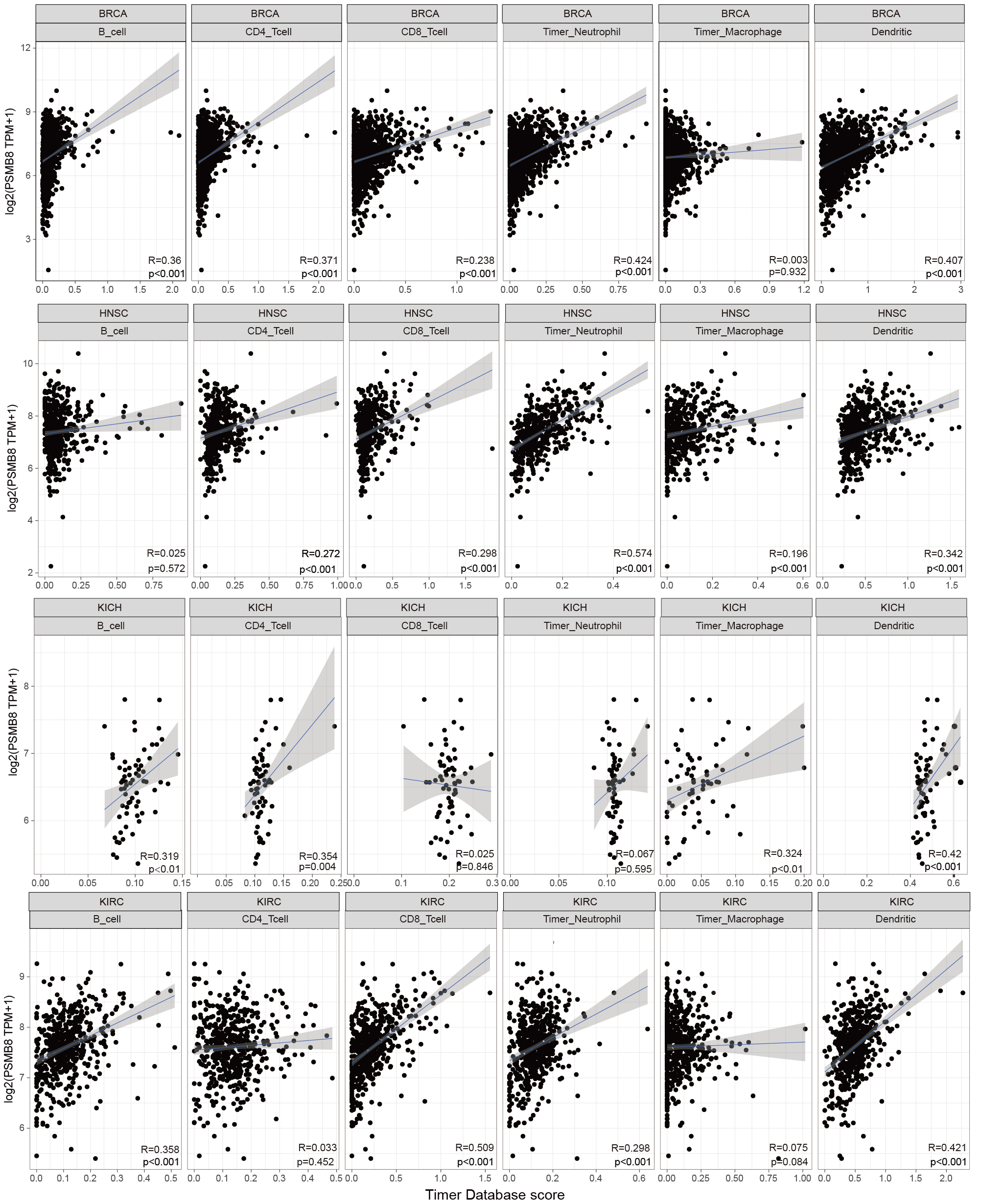


Supplementary Figure S4: Correlation between six immune cell inﬁltration scores and PSMB8 mRNA expression in KIRP, LCG, LIHC, and LUAD.


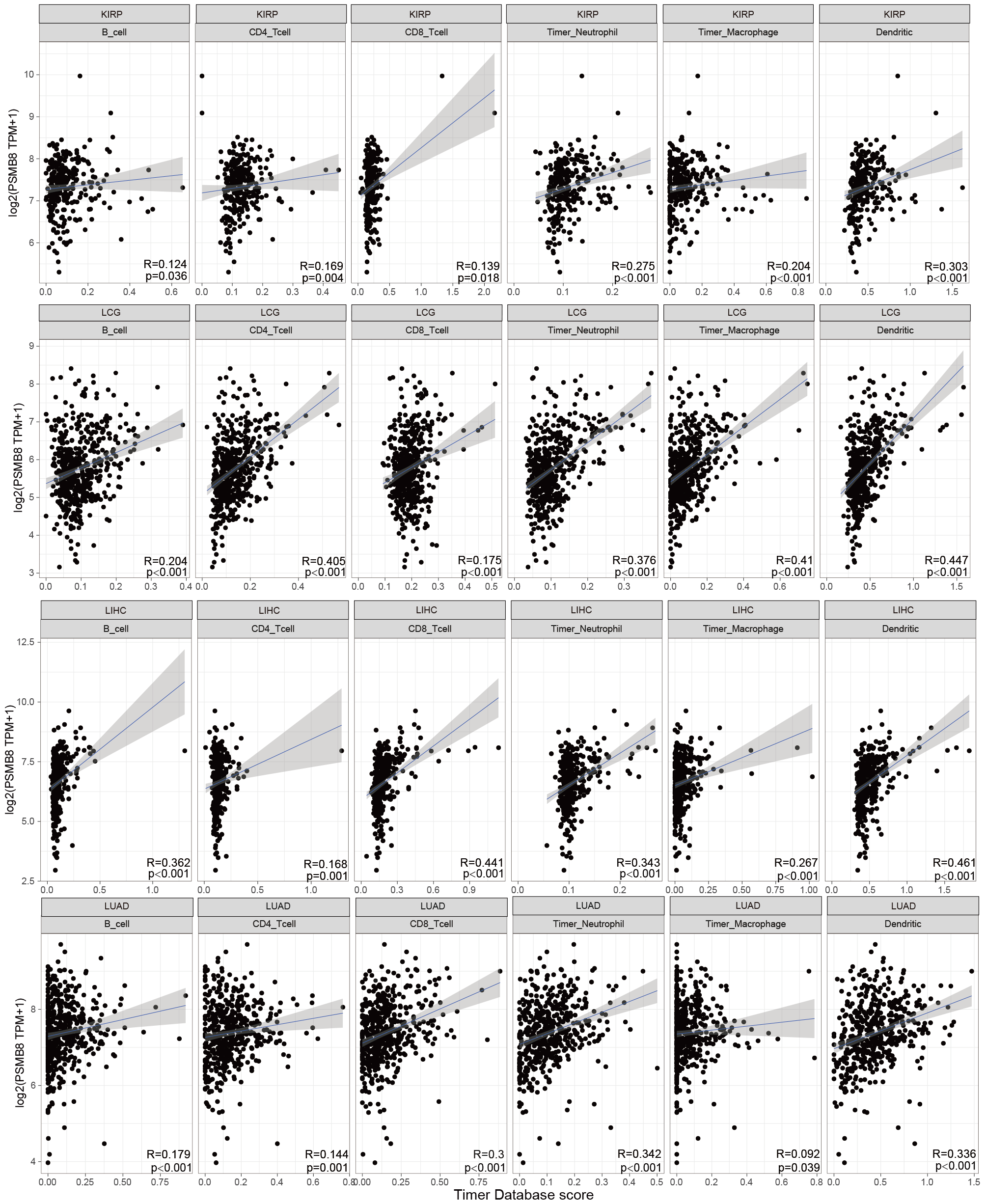


Supplementary Figure S5: Correlation between six immune cell inﬁltration scores and PSMB8 mRNA expression in LUSC, MESO, OV, and PAAD.


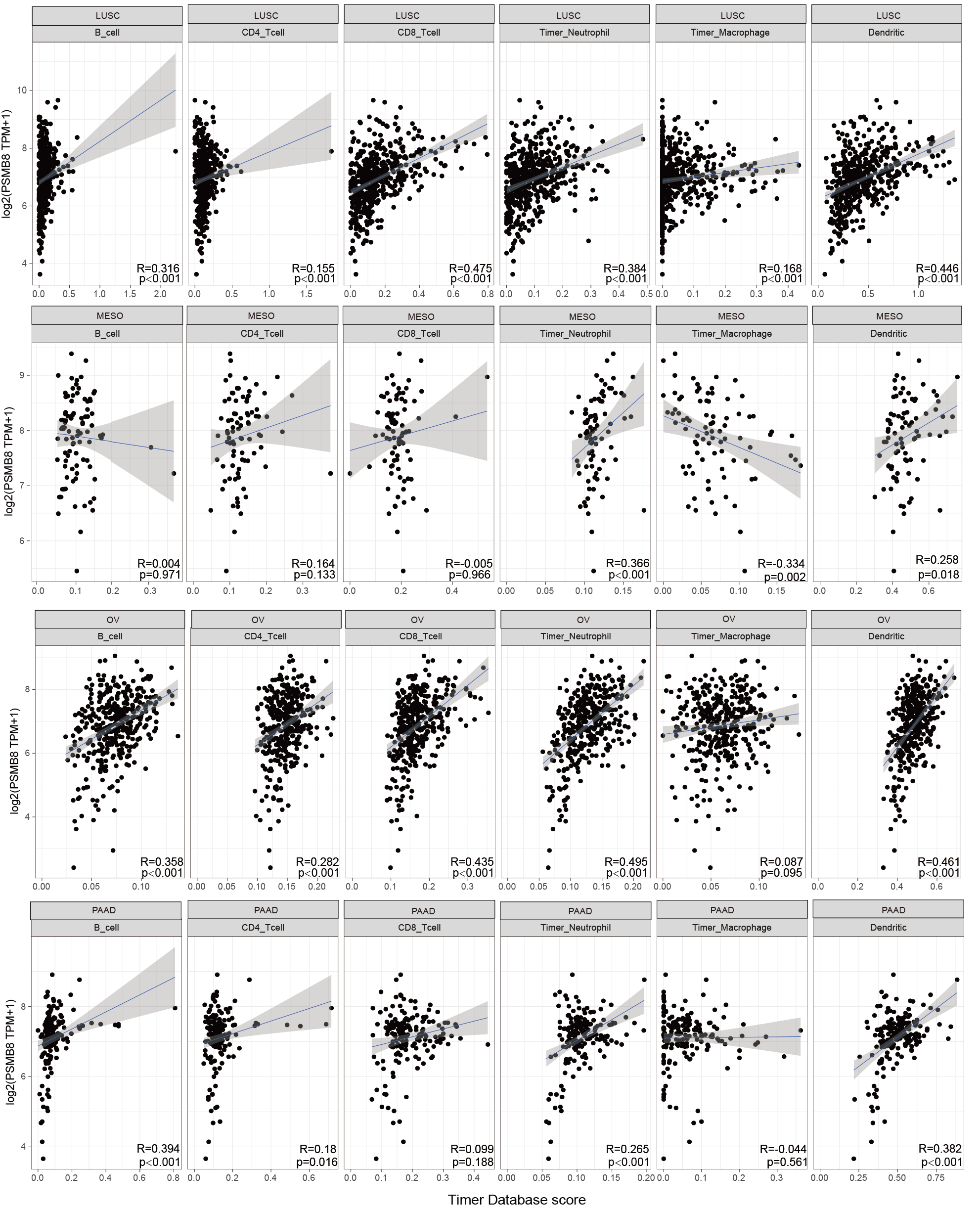


Supplementary Figure S6: Correlation between six immune cell inﬁltration scores and PSMB8 mRNA expression in PCPG, PRAD, READ, and SARC.
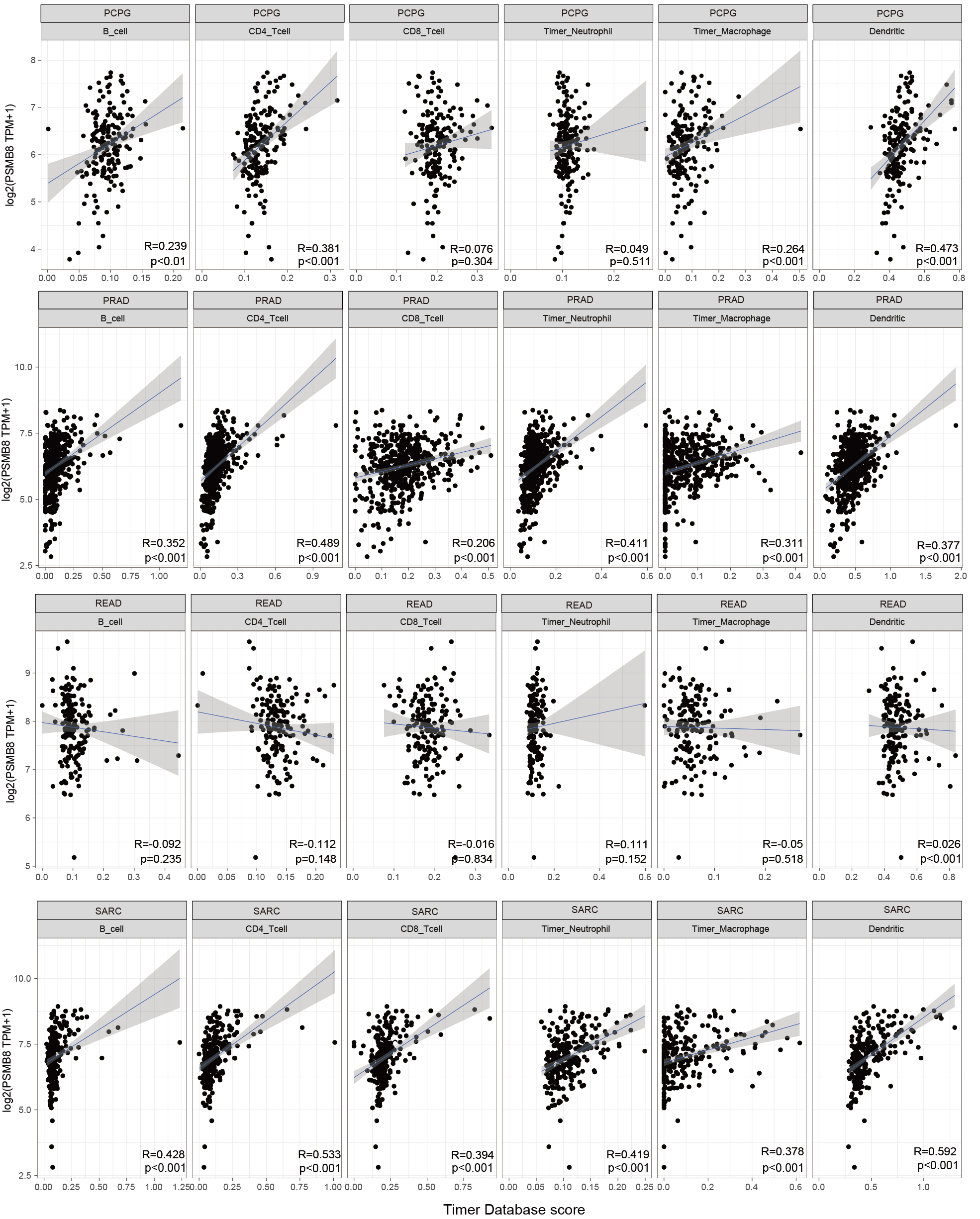


Supplementary Figure S7: Correlation between six immune cell inﬁltration scores and PSMB8 mRNA expression in SKCM, STAD, TGCT, and THCA.


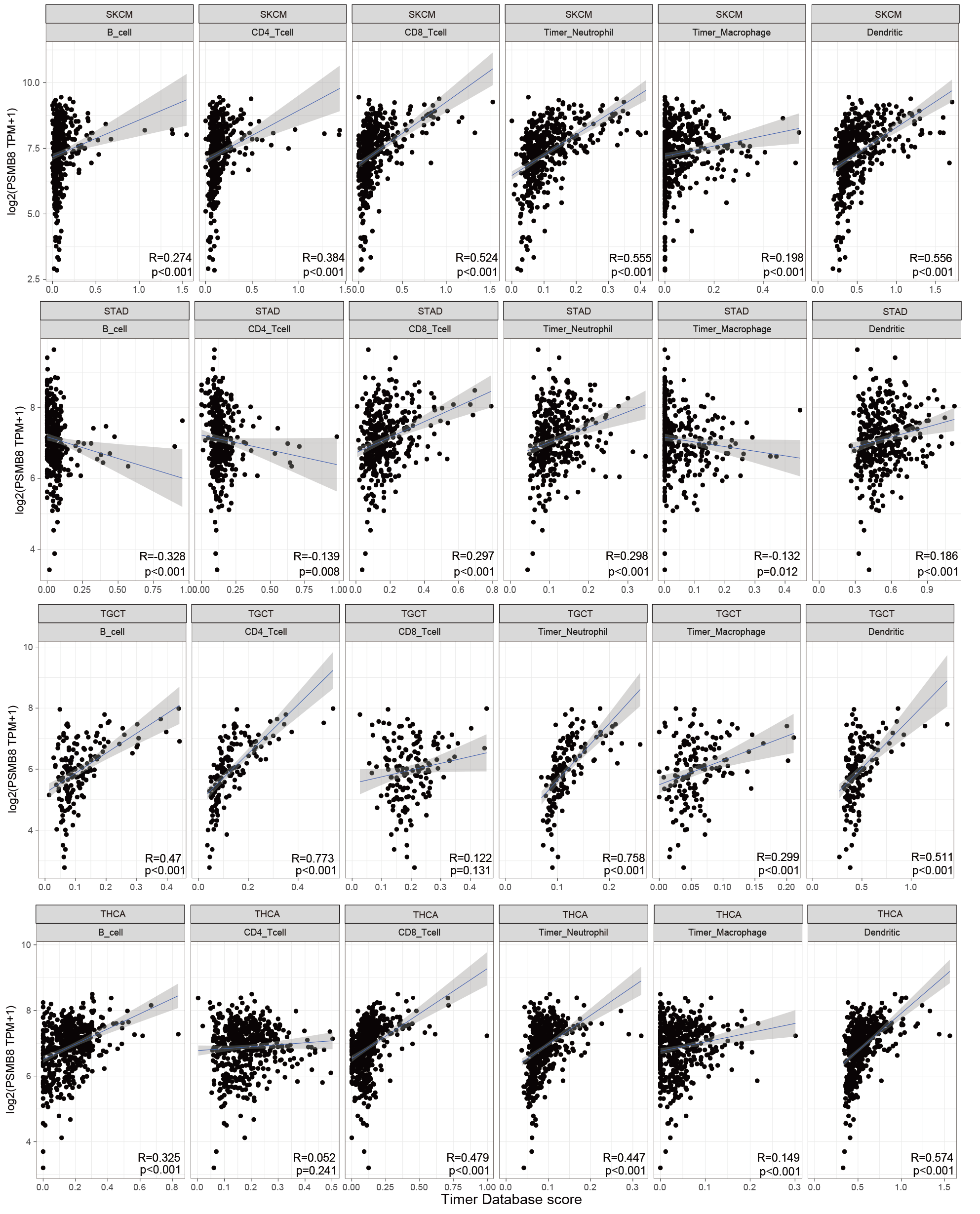


Supplementary Figure S8: Correlation between six immune cell inﬁltration scores and PSMB8 mRNA expression in THYM, UCEC, UCS, and UVM.


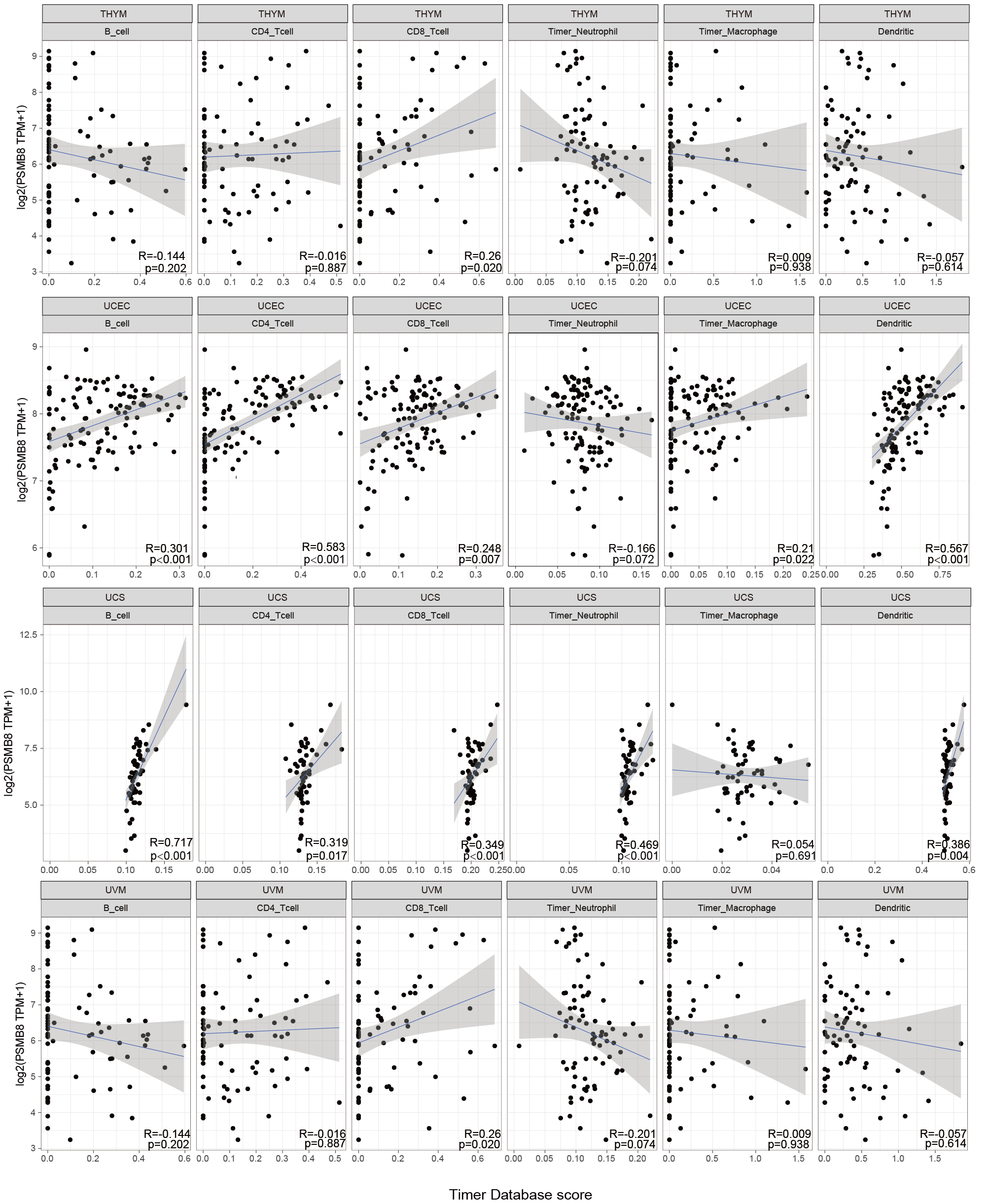

Supplement: Supplementary file 1 — Supplementary Information 1. [file 41598_2021_99724_MOESM1_ESM.docx]
